# Disordered eating behaviors in people with type 1 diabetes mellitus: a scoping review

**DOI:** 10.1590/1980-220X-REEUSP-2025-0177en

**Published:** 2025-12-08

**Authors:** Samara Jesus Sena Marques, Thiago Santos Garces, Samuel Miranda Mattos, Lara Lídia Ventura Damasceno, Açucena Leal de Araújo, Raphaelle Sampaio Campelo Forte, Kellen Alves Freire, Virna Ribeiro Feitosa Cestari, Consuelo Helena Aires de Freitas, Thereza Maria Magalhães Moreira

**Affiliations:** 1 Universidade Estadual do Ceará, Programa de Pós Graduação em Cuidado Clínicos em Enfermagem e Saúde, Fortaleza, CE, Brazil.; 2 Universidade Estadual do Ceará, Programa de Pós-Graduação em Saúde Coletiva, Fortaleza, CE, Brazil.; 3 Universidade Regional do Cariri, Crato, CE, Brazil.; 4 Centro Universitário Maurício de Nassau, Maracanaú, CE, Brazil.

**Keywords:** Diabetes Mellitus, Type 1, Feeding Behavior, Scoping Review

## Abstract

**Objective:**

To map disordered eating behaviors in people with Type 1 Diabetes Mellitus using the instrument *Diabetes Eating Problem Survey-Revised* (DEPS-R).

**Method:**

This is a scoping review, guided by JBI and in accordance with the PRISMA ScR recommendation. Four sources of information and grey literature were consulted. The search took place in January 2024. For the analysis of Similarity and Word Cloud, the software IRaMuTeQ was used. The interpretation of lexical analysis was based on the Theory of Social Representations.

**Results:**

The 21 articles selected describe the prevalence of disordered eating behaviors, with emphasis on insulin omission, binge eating, and dietary restriction. Body image, depressive symptoms, high HbA1c levels, and the presence of complications resulting from early high blood glucose levels are also related to disordered eating behaviors. The use of the DEPS-R instrument proved to be effective in detecting these behaviors.

**Conclusion:**

The results support the hypothesis that disordered eating behaviors are not just eating disorders in the traditional sense, but reflect dysfunctional coping strategies for chronic illness, often as attempts to control body weight or for emotional relief. Analytical studies on interdisciplinary clinical approaches using the DEPS-R instrument are fundamental to understanding the complexity of disordered eating behaviors.

## INTRODUCTION

Intensive insulin therapy management in type 1 Diabetes Mellitus (T1D) is performed continuously and requires commitment, planning, and constant monitoring from both the multidisciplinary team and the individuals with the disease. However, glycemic control is associated with weight gain, leading to the development of disordered eating behaviors (DEB), defined as unhealthy eating habits aimed at weight loss or control. These include insulin omission, known as diabulimia, binge eating, food restriction, and purgative methods^(1)^.

The main symptoms of eating disorders include the pursuit of an excessively thin body ideal, eating patterns characterized by calorie restriction and/or binge eating episodes, and the use of body weight and shape to determine self-esteem^(2)^. The scenario is even more worrying when considering the impacts of T1D on individuals’ self-esteem and mental health, especially among adolescents and young women, groups more susceptible to concerns about body image. People with T1D are at increased risk of developing symptoms of anxiety, depression, and body dissatisfaction, factors that, in turn, increase vulnerability to disordered eating behaviors^(3)^.

To identify these behaviors, the instrument *Diabetes Eating Problem Survey-Revised* (DEPS-R) was developed^(4,5)^, a specific self-report questionnaire to detect the risk of disordered eating behaviors in people with T1D. With a recommended cut-off score of 20 points or higher, DEPS-R facilitates further clinical assessment and individualization of the therapeutic plan.

However, despite the growing attention of the scientific community to the topic, including the construction and validation of the DESP-R, there still remains a significant gap in the identification of disordered eating behaviors in people with T1D, which highlights a lack of systematic evidence integrating the different forms of eating behaviors and their associated factors. This gap compromises the development of information on the topic, as well as the development of effective clinical guidelines and the continuing education of health professionals, aimed at enhancing prevention, early diagnosis, and comprehensive care for people with T1D.

In this context, conducting a scoping review presents itself as an appropriate methodological approach to map the current knowledge status, identify gaps, synthesize concepts, and guide future research. This approach is especially useful in emerging areas, such as the investigation of disordered eating behaviors in people with T1D, since the evidence is heterogeneous, dispersed, and often limited in scope and depth.

Thus, the objective of the study is to map disordered eating behaviors in people with type 1 Diabetes Mellitus found by the instrument DEPS-R to identify gaps in the interaction between the two outcomes and point out options for the development of future research on evidence-based therapies, in the identification of disordered eating behaviors associated with other factors.

## METHOD

### Design of Study

This is a scoping review, conducted in accordance with the standards established by the Joanna Briggs Institute (JBI). This time, it seeks to identify gaps in a given area of knowledge. To this end, the following steps were considered for preparation: 1) Definition and alignment of the objective(s) and question(s); 2) Development and alignment of inclusion criteria with the respective objectives and question; 3) Description of the planned approach for searching, selecting, extracting, analyzing, and presenting evidence; 4) Search for evidence; 5) Selection of evidence; 6) Extraction of evidence; 7) Analysis of results; 8) Presentation of results; and 9) Summary of the evidence in relation to the purpose of the review, conclusions, and implications of the findings^(6)^.

The protocol for this review was registered on the Open Science Framework (OSF) platform, with DOI: 10.17605/OSF.IO/SWNPE. To describe the search and selection process, in turn, the flowchart *Preferred Reporting Items for Systematic reviews and Meta-Analyses extension for Scoping Reviews* (PRISMA-ScR) was used^(7)^.

### Identifying the Guiding Question

The review question was structured based on the mnemonic Population, Concept and Context (PCC), where P refers to Population, that is, people with Type 1 Diabetes Mellitus; C, for concept, refers to disordered eating behaviors; and C for context, to the application of the instrument DEPS-R to identify disordered eating behaviors. The present study was guided by the following guiding question: What are the main disordered eating behaviors in people with type 1 Diabetes Mellitus, identified by the instrument DEPS-R?

### Eligibility Criteria

The inclusion criteria were articles addressing the applicability of the DEPS-R questionnaire for detecting disordered eating behaviors in people with T1D, published in any language or period. Studies in the design phase, editorials, or letters to the editor were excluded.

### Survey of Studies

For the vocabulary of controlled terms, the following were selected: “Diabetes mellitus type 1” and “Feeding behavior”, which are part of the Health Science Descriptors (DeCS), Medical Subject Headings (MeSH), and Embase Thesaurus (EMTREE). Furthermore, the uncontrolled terms “DEPS-R Instrument” and “DEPS-R” were considered, together with the Boolean operators AND and OR, as set out below in Chart 1.

**Chart 1:** Construction of the search strategy according to the PCC mnemonic – Fortaleza, CE, Brazil, 2024.

Stages	Acronym		
	P	C	C
Extraction	People with Type 1 Diabetes Mellitus	Disordered eating behaviors	Instrument DEPS-R
Conversion	*Type 1 Diabetes Mellitus*	*Feeding behavior*	DEPS-R
Combination	*Type 1 Diabetes Mellitus* Diabetes mellitus *Insulin Dependent Diabetes Mellitus*	*Feeding behavior* *Eating behavior* *Feeding related behavior* *Disturbed eating behaviors*	DEPS-R
Construction	*(“Diabetes mellitus type 1” OR “Diabetes Mellitus” OR “Insulin Dependent Diabetes Mellitus”)*	*(“Feeding behavior” OR “Eating behavior” OR “Feeding related behavior” OR “Disturbed eating behaviors”)*	DEPS-R
Use	*(“Diabetes mellitus type 1” OR “Diabetes Mellitus” OR “Insulin Dependent Diabetes Mellitus”) AND (“Feeding behavior” OR “Eating behavior” OR “Feeding related behavior” OR “Disturbed eating behaviors”) AND (DEPS-R)*		

The search took place in January 2024. The information sources used will be PubMed (National Library of Medicine), Excerpta Medica dataBASE (EMBASE), Web of Science, and Scopus. The information sources were accessed through the Federated Academic Community (CAFe) access to the Journals Portal of the Coordination for the Improvement of Higher Education Personnel (CAPES). The search in grey literature included Google Scholar and the CAPES Theses and Dissertations Catalog.

The selection of studies was conducted in three stages. The first stage involved identifying the articles by reading their title and abstract. The second stage, in turn, was the full reading of the selected articles, with the help of the software *Rayyan*, which controls the studies, allowing authors to select whether they are included or excluded in the respective stages^(8)^. At this stage, the authors used double-blinding to select the articles. To reduce conflicts of interest, a third reviewer was added to decide which articles would be chosen for the data extraction stage.

With the articles selected, we proceeded to the data extraction and coding stage of the selected articles according to title/coding, author/year of publication, journal, and general objective of the study, also considering the design, inclusion criteria, main disordered eating behaviors, and results.

### Data Analysis and Treatment

The articles that made up the sample were organized in a Microsoft Excel® spreadsheet. In addition, the software *Interface de R pour les Analyses Multidimensionnelles de Textes et de Questionnaires* (IRaMuTeQ) for Similitude and Word Cloud analysis. This software shows the lexical connections between words, revealing important relationships and central themes within speeches.

The similarity analysis, in turn, represents a network of words, where the nodes (central words) represent the most frequent and relevant terms in the texts analyzed. The lines between the nodes indicate co-occurrence, that is, terms that frequently appear together. The colors of the clusters suggest related themes or subthemes. In contrast, the word cloud is a visual representation of the frequency with which certain terms appear in the speeches/texts analyzed. The longer the word, the greater its frequency or relevance in the textual corpus. This visualization helps identify the main themes, concerns, and linguistic patterns of the participants or documents analyzed^(9)^.

The interpretation of lexical analysis was based on the Theory of Social Representations^(10)^, which understands that common knowledge is formed from shared social experiences, influencing attitudes, practices and perceptions. This theory was particularly useful in understanding how people with T1D construct meanings around body image, insulin use, and diet. Furthermore, specific references to eating disorders in people with T1D were considered, such as the practice of omitting insulin for weight loss purposes^(11)^.

## RESULTS

**Figure 1:**
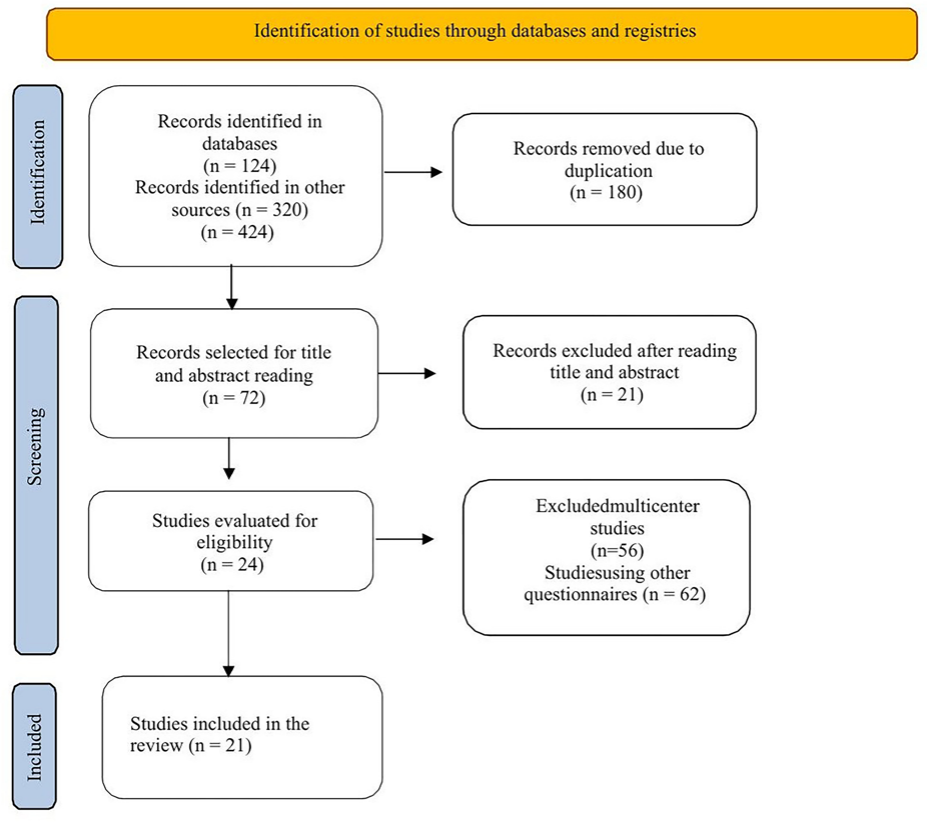
PRISMA 2020 flow diagram for new systematic reviews that included database and registry searches – Fortaleza, Ceará, Brazil, 2024.

A total of 124 articles were found in the databases, added to the 320 in the grey literature, totaling 424 articles. Of these, only 21 articles comprised the final sample, see Figure 1.

Source^(7)^.

The articles in the sample mostly have a cross-sectional methodological design, highlighting epidemiological measures, such as the prevalence of disordered eating behaviors in this population, based on validated instruments such as the DEPS-R. The main findings are organized in Chart 2 below.

**Chart 2:** Characterization and coding of the sample according to title, author, year of publication, journal and general objective – Fortaleza, Ceará, Brazil, 2024.

Title/Encoding	Author	Journal	General objective
*Diabetes-specific eating disorder and possible associated psychopathologies in adolescents with type 1 diabetes mellitus/A1*	Gürkan Tarçın^(12)^	Eat and Weight Disorders	To investigate the prevalence of risk of disordered eating behaviors in adolescents with type 1 diabetes mellitus and to examine associated psychopathies.
*The Diabetes Eating Problem Survey-Revised (DEPS-R) in a Greek Adult Population with Type 1 Diabetes Mellitus: Model Comparison Supporting a Single Factor Structure/A2*	Calliope Karastogiannidou^(13)^	Nutrients	To validate the factor structure of the Greek version of the DEPS-R using confirmatory factor analysis; to investigate the reliability and convergent validity of the Greek version of the DEPS-R; and to compare a single-factor DEPS-R model with multiple-factor models.
*Eating disorders between male and female adolescents with type 1 diabetes mellitus in Korea/A3*	Hye-Ryeon Park^(14)^	Belitung Nursing Journal	To identify sex-specific factors that influence eating disorders in adolescents with type 1 diabetes.
*Assessment of eating disorders with the diabetes eating problems survey – revised (DEPS-R) in a representative sample of insulin-treated diabetic patients: a validation study in Italy/A4*	Frederica Pinna^(15)^	BMC Psychiatry	To evaluate the psychometric characteristics of the Italian version of the DEPS-R scale in a sample of diabetic patients treated with insulin.
*Reliability and validity of the Diabetes Eating Problem Survey in Greek adults with type 1 diabetes mellitus/A5*	Apergi^(16)^	Psychiatriki	To translate and adapt the DEPS-R questionnaire into Greek so that it can be used as a short, specific, self-administered screening tool for the detection of eating disorders in people with type 1 diabetes mellitus.
*Disturbed Eating Behavior and Omission of Insulin in Adolescents Receiving Intensified Insulin Treatment: A nationwide population-based study/A6*	Line Wisting^(17)^	Diabetes Care	To establish the prevalence of eating disorders and insulin omission among adolescents with type 1 diabetes undergoing intensive insulin treatment in a nationwide population-based study.
*Associations between disordered eating behavior, diabetes distress and emotion regulation strategies in adults with type 1 diabetes: Results from a Dutch-Italian cross-sectional study/A7*	Jiska Embaye^(18)^	Diabetic medicine	To examine the associations between disordered eating behaviors, diabetes distress, and emotion regulation strategies in the context of type1 diabetes mellitus.
*Psychometric properties and factor structure of the diabetes eating problem survey-revised (DEPS-R) among adults with type 1 diabetes mellitus/A8*	Yasemin Atik-Altınok^(19)^	Eat and Weight Disorders	To examine the factor structure of the Turkish version of the DEPS-R in adults with T1D and its internal consistency and construct validity.
*Emotional eating and disordered eating behaviors in children and adolescents with type 1 diabetes/A9*	Carlo Ripoli(20)	Scientific Review	To assess whether, in children and adolescents with T1D, the presence of emotional eating is associated with metabolic control (glycated hemoglobin, plasma lipids, uric acid), demographic data (age, sex, duration of diabetes), anthropometric data (BMI-SDS), therapeutic data (method of insulin administration, with pump or multiple daily injections and total daily dose) and clinical variables.
*Prevalence of Eating Behaviors and Their Influence on Metabolic Control of Children with Type 1 Diabetes Mellitus/A10*	Samaneh Farnia(21)	Journal of Pediatrics Review	To explore the prevalence of eating behaviors and their influence on the metabolic control of children with type 1 diabetes mellitus.
*Validation of the traditional Chinese version of the diabetes eating problem survey-revised and study of the prevalence of disordered eating patterns in Chinese patients with type 1 DM/A11*	Lok Whi Ching^(22)^	BMC Psychiatry	To develop and validate the traditional Chinese version of the DEPS-R (C-DEPS-R), examining the reliability and validity of the scale in adults and adolescents with type 1 diabetes mellitus who attended specialized diabetes mellitus care.
*High prevalence of disordered eating behavior in Danish children and adolescents with type 1 diabetes/A12*	Francisca Nilsson^(23)^	Pediatric Diabetes	To determine the prevalence of disordered eating behaviors in a Danish cohort of children and adolescents with type 1 Diabetes Mellitus aged 11 to 19 years and to characterize them regarding metabolic control and relevant clinical data.
*Translation and validation of the Diabetes Eating Problem Survey to screen eating disorders in patients with type-1 diabetes mellitus/A13*	Cintia Sancanato^(24)^	Medicine Clinic	To translate and validate, for the Spanish population, a specific questionnaire designed to detect the risk of an eating problem in a sample of individuals with type 1 Diabetes Mellitus.
*Disordered eating behaviors in adolescents with type 1 diabetes: A cross-sectional population-based study in Italy/A14*	Valentino Cherubini^(25^)	International Journal of Eating Disorders	To evaluate the association of clinical, metabolic, and socioeconomic factors with eating disorder behaviors among adolescents with type 1 diabetes screened using the Diabetes Eating Problem Survey-Revised (DEPS-R).
*Hypothalamic Gliosis in Adolescents with Type 1 Diabetes and Disordered Eating Behaviors/A15*	Angel Siu Ying Nip^(26)^	Journal of the Endocrine Society	To determine the feasibility of evaluating hypothalamic gliosis with structural magnetic resonance imaging in adolescents with Type 1 Diabetes Mellitus with and without disordered eating behaviors.
*Disordered Eating Behaviors and Insulin Restriction in Saudi Adolescents and Young Adults with Type 1 Diabetes/A16*	Saeed Yafei^(27)^	Medicine	To establish the prevalence of disordered eating behaviors and associated clinical features in adolescents and young adults with Type 1 Diabetes Mellitus, and the impact of disordered eating behaviors in glycemic parameters.
*Disturbed Eating Behaviors in Adolescents and Emerging Adults With Type 1 Diabetes: A One-Year Prospective Study/A17*	Koen Luyckx^(28)^	Diabetes Care	Examined disordered eating behaviors over a one-year period and investigated the directionality of effects linking disordered eating behaviors to the specific functioning of diabetes and depressive symptoms in adolescents and emerging adults.
*Brief Screening Tool for Disordered Eating in Diabetes/A18*	Markowitz^(4)^	Diabetes Care	To update and validate a diabetes-specific screening tool for eating disorders (DEPS) in contemporary youth with type 1 diabetes.
*Prevalence of disordered eating in adults with type 1 diabetes in an Australian metropolitan hospital/A19*	Arleen Watt^(29)^	Health and social care in the Community	To determine the prevalence of disordered eating behaviors and establish their identifiable factors in adults with type 1 diabetes attending a tertiary hospital service.
*Disordered eating behaviours and body shape dissatisfaction among adolescents with type 1 diabetes: a cross-sectional study/A20*	Lidya Daniel^(30)^	Journal of Eating Disorders	Assess the magnitude of the disordered eating behaviors and its relationship with body shape dissatisfaction among adolescents with diabetes being monitored.
*A Risk Profile for Disordered Eating Behaviors in Adolescents with Type 1 Diabetes: A Latent Class Analysis Study/A21*	Giada Boccolini^(31)^	Nutrients	To identify a risk profile for disordered eating behaviors in young people with type 1 diabetes based on their dietary intake, lipid profile, body mass index, and glycometabolic control.

DEPS-R: Diabetes Eating Problem Survey-Revised.

Studies highlight the presence of multiple disordered eating behaviors among people with T1D. Among these, insulin omission was the most prevalent^(12,13,14,15,16,17,18,19,20,21,22,23,24,25,26,27,28,29,30,31,32)^, along with the inappropriate use of insulin^(16,17,18,19,20)^, binge eating^(12,14,20,30)^, dietary restriction^(12,15,16,23,24)^, and bulimic behaviors^(22,25)^ (see Chart 3). Additionally, the presence of disordered eating behaviors in this population was associated with factors related to body image, such as high Body Mass Index (BMI), dissatisfaction with body image, and symptoms of depression, distress, and anxiety. In addition to this, the presence of high HbA1c levels and of complications resulting from early high glycemic levels was highlighted.

**Chart 3:** Methodological characterization and main findings of studies on disordered eating behaviors in people with type 1 diabetes – Fortaleza, Ceará,Brazil, 2024.

Codification	Design	Inclusion criteria	Disordered eating behaviors	Results
A1	Cross-sectional study	Adolescents aged 12 to 18 years, diagnosed with type 1 diabetes for at least one year.	Insulin omission, binge eating, dietary restriction.	The emergence of disordered eating behaviors is related to body image dissatisfaction and high body mass index.
A2	Cross-sectional study	Age over 18 and diagnosed with type 1 diabetes.	Binge eating, food restriction.	The DEPS-R scale had good reliability and was positively associated with glycated hemoglobin levels.
A3	Secondary data analysis study	Patients with Diabetes mellitus over 18 years of age.	Insulin omission, binge eating.	Disordered eating behaviors are associated with body image dissatisfaction and depression.
A4	Cross-sectional study	Diabetic patients treated with insulin, between 13 and 55 years old.	Insulin omission, dietary restriction, purgative behaviors.	Women had higher scores on the DEPS-R, a higher percentage of at least one diagnosis of Disordered Eating Behavior.
A5	Cross-sectional study	Age over 18, diagnosed with type 1 diabetes for more than a year.	Binge eating, inappropriate insulin use.	Factor analysis revealed four factors (diet, weight loss, insulin use, and compensatory behaviors).
A6	Cross-sectional study	People diagnosed with type 1 diabetes aged 11 to 19 years.	Insulin omission, binge eating.	The presence of disordered eating behaviors in people with type 1 diabetes is associated with high values of body mass index, glycated hemoglobin, and pubertal stage.
A7	Cross-sectional study	People diagnosed with type 1 diabetes who are 18 years of age or older.	Insulin omission, binge eating.	Path analysis, with small and medium effect sizes, revealed that more diabetes distress was associated with more eating disorders.
A8	Cross-sectional study	People diagnosed with type 1 diabetes between the ages of 18 and 55.	Binge eating, food restriction.	Analysis of DEPS-R results correlated significantly with body mass index.
A9	Cross-sectional study	People diagnosed with type 1 diabetes.	Binge eating, emotional eating.	Individuals with DEB have higher emotional eating scores than individuals without DEB.
A10	Cross-sectional study	People diagnosed with type 1 diabetes.	Omission of insulin, dietary restriction.	Children and adolescents exhibit behavior of avoiding insulin doses.
A11	Cross-sectional study	Patients with diabetes using insulin, diagnosed in the last six months.	Insulin omission, binge eating.	DEPS-R has good consistency. Women with type 1 diabetes have higher scores and in this group the disordered eating behaviors are associated with symptoms of depression.
A12	Cross-sectional study	People with type 1 diabetes with duration of illness of more than a year.	Binge eating, food restriction.	Disordered eating behaviors are associated with high values of glycated hemoglobin and body mass index.
A13	Cross-sectional study	Diagnosis of type 1 diabetes for at least 12 months, in a phase of clinical stability and on insulin treatment.	Binge eating, bulimic behavior, dietary restriction.	Factor analysis of the DEPS-R resulted in five factors: Eating attitudes; Bulimic behavior; Weight control; Avoidance; and Restriction.
A14	Cross-sectional study	Type 1 diabetes diagnosed at least one year before the start of the study.	Insulin omission, binge eating.	A DEPS-R-positive clinical profile was identified: excess weight, little time spent in physical activity, low socioeconomic status, poor metabolic control, skipping insulin doses. Furthermore, overweight youth were six times more likely to be DEPS-R positive.
A15	Cross-sectional study	Teenagers with Type 1 diabetes aged 13 to 19 years.	Binge eating, Insulin omission.	People with Diabetes Mellitus and disordered eating behaviors have high levels of glycated hemoglobin.
A16	Cross-sectional study	Saudi adolescents and young adults with Type 1 diabetes (aged 12 to 25 years) on multiple insulin treatments for at least one year.	Insulin omission, binge eating.	Participants with disordered eating behaviors had higher glycated hemoglobin and body mass index compared to those without.
A17	Cross-sectional study	Dutch-speaking young people, aged 16–28, with and without type 1 diabetes and cognitive impairment.	Insulin omission, binge eating.	People with type 1 diabetes who have disordered eating behaviors have depression and high values of glycated hemoglobin.
A18	Cross-sectional study	Teenagers with Type 1 diabetes with a diagnosis time of one year.	Binge eating, Insulin omission.	The DEPS-R showed positive correlations with body mass index, family conflict, and negative affect of young people around blood glucose monitoring.
A19	Cross-sectional study	Patients with Type 1 diabetes aged 18 to 65 years with a diagnosis of the disease for at least one year.	Insulin omission, binge eating.	Women with type 1 diabetes have high glycated hemoglobin and body mass index values.
A20	Cross-sectional study	Adolescents with diabetes (10 to 19 years old) being monitored at the diabetes center of five public hospitals.	Binge eating, Insulin omission.	Identified as associated with disordered eating behaviors, factors such as dissatisfaction with body image, excess weight and diabetic complications.
A21	Multicentric study	Adolescent with Type 1 diabetes and with a diagnosis time of at least one year.	Binge eating, Insulin omission.	Adolescents at risk for developing disordered eating behaviors have a high body mass index.

DEPS-R: Diabetes Eating Problem Survey-Revised.

**Figure 2:**
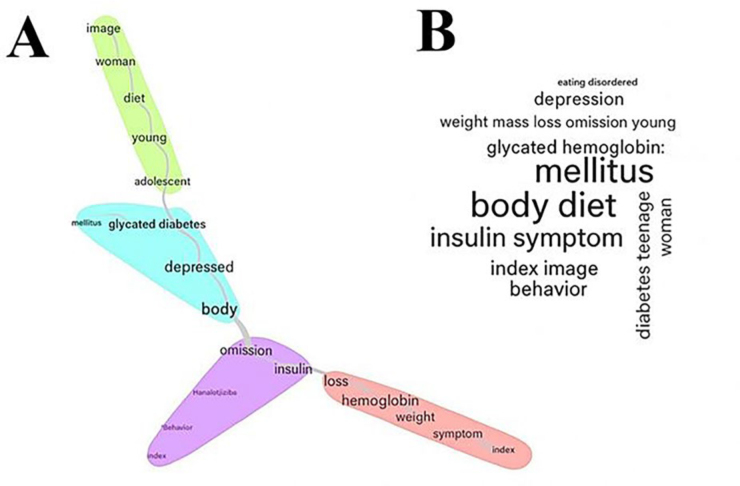
Similarity analysis (A) and word cloud (B) of terms related to disordered eating behaviors in people with type 1 diabetes mellitus.

Below, in Figure 2A, the similarity analysis technique shows the lexical connections between words associated with disordered eating behaviors in people with T1D. A central nucleus is highlighted in purple bold by the terms omission and insulin, referring to the centrality of the habit of insulin omission within disordered eating behaviors in this population. This association suggests that it indicates intentional risk behavior, often for weight control, characteristic of eating disorders such as diabulimia.

Furthermore, the group of words highlighted in blue refers to the words “depressive”, “glycated” and “body” and “hemoglobin”, making an association with mental health, such as the prevalence of depressive disorders in people with T1D, associated with glycemic control (glycated hemoglobin) and body image, similar to the green group, which points to the most vulnerable population profile: adolescents and young females, who are more likely to develop eating disorders due to the influence of body image and diets. Finally, the group in red shows the physical consequences of disordered eating behaviors: such as “loss”, “weight”, “mass index”, and “hemoglobin”.

In Figure 2B, a graphical representation of the word cloud, the terms “hemoglobin”, “body”, “glycated”, “insulin” and “weight” represent the central axes of the analysis and relate to topics such as glycemic control, body weight, and body image, strongly related to eating disorders, in addition to insulin administration and its possible omission, with relevant implications for the maintenance of disordered eating behaviors in people with T1D.

## DISCUSSION

Studies indicate that individuals with T1D often present with diabulimia, binge eating, and restriction, revealing the complexity of nutritional management in this population, requiring an approach beyond traditional dietary recommendations. These behaviors, often associated with body image dissatisfaction, depressive symptoms, and anxiety, reinforce the need for nutritional strategies that consider the emotional and psychosocial determinants of eating. Furthermore, the relationship between these dietary patterns and poor glycemic control highlights the relevance of individualized nutritional interventions, integrated with a multidisciplinary team, aiming to prevent complications early in this population.

In this regard, studies have underscored the high prevalence of disordered eating behaviors in people with T1D, with rates ranging from 30% to 60% of cases, distributed with greater emphasis among young women and adolescents^(32,33,34)^. In Brazil, it is estimated that 45% of adolescents with T1D had high scores for risk of eating disorders, with a statistically significant association between these behaviors and inadequate glycemic control. These findings indicate that disordered eating behaviors are not isolated manifestations, but are deeply integrated into the experience of living with T1D, influencing both physical and psychological well-being^(35)^.

### Omission of Insulin

Insulin omission is the most common disordered eating behavior among people with T1D, as intentionally induced glycosuria causes weight loss. In parallel, there is the presence of binge eating, especially in moments of emotional stress, followed by extreme restrictions as a form of compensation. Its causes are mainly associated with consequent weight loss. Additionally, there are other possible reasons for insulin reduction or omission, including injection anxiety, fear of hypoglycemia, interference with activities of daily living, or as a result of diabetes burnout due to lack of adherence to treatment^(13,22,35)^.

In young women, the practice of insulin omission has been reported in up to 40%, so that this population has 2.4 times more risk of eating disorders than women of the same age without DM^(36)^. This panorama reflects the complex interaction between the metabolic aspects of the disease and psychosocial factors, hormonal issues, concern with body image, aesthetic pressure, and weight dissatisfaction.

Puberty and adolescence are particularly critical periods, as they involve physiological and cognitive changes that intensify vulnerability to the development of dysfunctional eating behaviors^(15,35)^. Furthermore, emotional factors such as anxiety, depression, stress and low self-esteem have been identified as relevant triggers for the manifestation of these behaviors^(13,26)^. Thus, disordered eating behaviors in individuals with T1D represent a multifactorial clinical condition that requires an interdisciplinary approach and early detection strategies.

Moreover, adolescents are more likely to consume foods with a lipid profile, which contributes to an increase in BMI, a risk factor for the development of disordered eating behaviors. This time, young people with a longer time since diagnosis, higher body weight and high HbA1c levels appear to endorse disordered eating behaviors^(22,32)^.

In this context, neurobiology also plays an important role. The hypothalamus and brainstem are responsible for regulating appetite and weight. Inflammation of this organ influences the emergence and maintenance of eating-related behaviors, as well as disordered eating behaviors, including insulin omission and binge eating in individuals with T1D^(12,18,36)^.

Studies show that distorted body image and the pursuit of thinness are factors associated with the omission of insulin as a weight control method, a practice known as diabulimia^(11,16,34)^. This practice may seem functional in the short term due to weight loss associated with induced hyperglycemia, but it is directly linked to worse metabolic outcomes and a higher risk of chronic complications^(20)^. Body dissatisfaction, in turn, is associated with increased BMI, which feeds back into the cycle of aesthetic concerns, insulin deficiency, and dietary dysregulation^(36)^.

### Food Restriction and Binge Eating

It was evident that dissatisfaction with body image and the use of unhealthy diets are factors that contribute to the emergence of disordered eating behaviors, negatively affecting glycemic control and the mental health of patients, as well as the practice of dietary restriction^(13,24,26)^.

Binge eating is sometimes part of a vicious cycle of dieting that involves the excessive consumption of foods with a high glycemic value that, when combined with the absence of insulin, promotes weight loss through osmotic diuresis, glycosuria, ketonuria, dehydration, and also allows the excretion of energy obtained from food through urine^(19)^.

In addition to the issue of body image, for some individuals, meals are considered resources for dealing with negative emotions. Individuals with T1D have a higher prevalence of depression and anxiety compared to the general population, which can be explained by both biological factors and the psychological burden of ongoing self-care^(13,26)^. This condition, understood as emotional eating, is directly related to deficient metabolic control and also to the appearance of anxious and depressive symptoms, which culminate in episodes of binge eating followed by guilt and compensatory behaviors, such as food restriction or insulin omission^(36,37)^. Studies suggest that high levels of glycated hemoglobin (HbA1c) are associated not only with poor therapeutic adherence, but also with the presence of depressive symptoms and a worse perception of body image, forming a complex cycle between metabolic health and mental health^(4,12)^.

Furthermore, the emotional burden gives binge eating the search for foods that can alleviate negative feelings experienced, especially by people of younger age and higher BMI^(7)^. In view of this, the early detection of disordered eating behaviors through the use of validated questionnaires, such as the DEPS-R, suggests a more careful evaluation of eating habits and lifestyle practices, thus favoring a patient-centered approach, to facilitate the construction of an effective therapeutic bond and the implementation of individualized interventions, adjusted to the individuals’ reality^(13,15,20)^.

Research highlights that high DEPS-R scores are strongly associated with high HbA1c levels, higher BMI, and symptoms of anxiety and depression, emphasizing the interrelationship between mental health, body self-perception, and treatment adherence^(20,34,35,38)^. Thus, DEPS-R represents not only a diagnostic tool, but a strategic resource in the integrated management of health care for people with T1D.

Despite advances in identifying and understanding disordered eating behaviors in people with T1D, there are still important gaps that need to be explored in future studies. It is essential to develop longitudinal research that allows us to understand the trajectories of disordered eating behaviors over time and their relationship with glycemic control, emotional state, and clinical outcomes. Additionally, there is a lack of studies addressing integrated interventions, focusing on mental health, nutritional education, and treatment adherence, especially in low- and middle-income contexts, such as Brazil.

Likewise, research using qualitative approaches can contribute to a deeper understanding of the subjective experiences and barriers faced by these individuals in managing their diet and insulin. In this sense, future studies could strengthen the development of early screening protocols and personalized therapeutic interventions, promoting more humanized, effective, and interdisciplinary care for people with T1D.

There are also limitations that must be recognized. Most of the studies included used self-report methods to assess disordered eating behaviors, which may introduce response biases and underreporting of behaviors. Furthermore, variability in diagnostic criteria and assessment tools across studies may have impacted the comparability of results.

Furthermore, although systematic approaches were adopted for the inclusion of studies, the possibility of selection bias cannot be completely ruled out. However, these limitations were partially mitigated by the application of strict inclusion criteria and critical analysis of the methodologies used in the selected studies. The geographic diversity of the samples also strengthens the generalizability of the findings, providing a comprehensive view of disordered eating behaviors in populations with T1D.

## CONCLUSION

The study showed that disordered eating behaviors are common in people with T1D, with insulin omission standing out as the most prevalent behavior, followed by binge eating and food restriction. The review highlighted that these behaviors are influenced by a complex interaction of emotional, cognitive, and metabolic factors, such as dissatisfaction with body image, as well as elevated HbA1c and BMI levels. These findings support the hypothesis that disordered eating behaviors are not just eating disorders in the traditional sense, but reflect dysfunctional coping strategies for chronic illness, often used as an attempt to control body weight or for emotional relief.

Additionally, it was observed that the individuals most vulnerable to these behaviors are adolescents and young women, groups that face greater challenges regarding acceptance of the diagnosis, bodily changes, and the demands of intensive insulin treatment. Intentional insulin omission, often motivated by a desire to lose weight, may initially appear effective for weight loss, but is associated with unfavorable outcomes, such as poor glycemic control. These data highlight the importance of interdisciplinary clinical approaches, as well as early screening strategies, such as the use of DEPS-R, to mitigate the impacts of these behaviors on the physical and mental health of people with T1D.

## DATA AVAILABILITY

The data is available on OSF: https://www.doi:10.17605/OSF.IO/SWNPE.
