# Supplementary material for: Disordered eating behaviors in people with type 1 diabetes mellitus: a scoping review
Source: Rev Esc Enferm USP. 2025 Dec 8;59:e20250177. doi: 10.1590/1980-220X-REEUSP-2025-0177en (PMC12698104; doi:10.1590/1980-220X-REEUSP-2025-0177en)
Supplement: Translation

# Translation: Comportamentos alimentares desordenados em pessoas com diabetes mellitus tipo 1: revisão de escopo

**DOI:** 10.1590/1980-220X-REEUSP-2025-0177pt

**Authors:** Samara Jesus Sena Marques, Thiago Santos Garces, Samuel Miranda Mattos, Lara Lídia Ventura Damasceno, Açucena Leal de Araújo, Raphaelle Sampaio Campelo Forte, Kellen Alves Freire, Virna Ribeiro Feitosa Cestari, Consuelo Helena Aires de Freitas, Thereza Maria Magalhães Moreira

**Affiliations:** 1 Universidade Estadual do Ceará, Programa de Pós Graduação em Cuidado Clínicos em Enfermagem e Saúde, Fortaleza, CE, Brasil.; 2 Universidade Estadual do Ceará, Programa de Pós-Graduação em Saúde Coletiva, Fortaleza, CE, Brasil.; 3 Universidade Regional do Cariri, Crato, CE, Brasil.; 4 Centro Universitário Maurício de Nassau, Maracanaú, CE, Brasil.

**Keywords:** Diabetes Mellitus Tipo 1, Comportamento Alimentar, Revisão de escopo

## Abstract

**Objetivo:**

Mapear os comportamentos alimentares desordenados em pessoas com Diabetes Mellitus tipo 1 a partir do instrumento *Diabetes Eating Problem Survey-Revised* (DEPS-R).

**Método:**

Trata-se de uma revisão de escopo, orientada pelo JBI e de acordo com a recomendação do PRISMA ScR. Foram consultadas quatro fontes de informação e literatura cinzenta. A busca ocorreu no período de janeiro de 2024. Para análise de Similitude e Nuvem de Palavras foi utilizado o software IRaMuTeQ. A interpretação da análise lexical foi embasada na Teoria das Representações Sociais.

**Resultados:**

Os 21 artigos selecionados descrevem a prevalência de comportamentos alimentares desordenados, com destaque para a omissão de insulina, compulsão alimentar e restrição alimentar. A imagem corporal, sintomas depressivos, alta taxa HbA1c e a presença de complicações decorrentes de altos níveis glicêmicos de forma precoce também estão relacionados aos comportamentos alimentares desordenados. A utilização do instrumento DEPS-R mostrou-se eficaz na detecção desses comportamentos.

**Conclusão:**

Os resultados sustentam a hipótese de que os comportamentos alimentares desordenados não são apenas distúrbios alimentares no sentido tradicional, mas refletem estratégias disfuncionais de enfrentamento da doença crônica, muitas vezes como tentativa de controle de peso corporal ou de alívio emocional. Estudos analíticos sobre abordagens clínicas interdisciplinares utilizando o instrumento DEPS-R são fundamentais para a compreensão da complexidade dos comportamentos alimentares desordenados.

## INTRODUÇÃO

O gerenciamento intensivo da insulinoterapia no Diabetes Mellitus tipo 1 (DM1) é desempenhado de forma contínua e demanda comprometimento, planejamento e monitoramento constante tanto da equipe multiprofissional quanto dos indivíduos com a doença. No entanto, o controle glicêmico está associado ao ganho de peso, propiciando o desenvolvimento de comportamentos alimentares desordenados, definidos como hábitos alimentares não saudáveis, visando perda ou controle de peso. Dentre estes, estão incluídos a omissão de insulina, que recebe o nome de diabulimia, a compulsão alimentar, a restrição de alimentos e os métodos purgativos^(1)^.

Os principais sintomas do transtorno alimentar incluem a busca por um ideal de corpo excessivamente magro, padrões alimentares caracterizados por restrição calórica e/ou episódios de compulsão alimentar e uso do peso e forma corporal para determinar a autoestima^(2)^. O cenário é ainda mais preocupante quando seconsideram os impactos do DM1 sobre a autoestima e a saúde mental dos indivíduos, especialmente entre adolescentes e mulheres jovens, grupos mais suscetíveis a preocupações com imagem corporal. Pessoas com DM1 apresentam maior risco de desenvolver sintomas de ansiedade, depressão e insatisfação corporal, fatores que, por sua vez, aumentam a vulnerabilidade a comportamentos alimentares desordenados^(3)^.

Para identificar esses comportamentos, foi desenvolvido o instrumento *Diabetes Eating Problem Survey-Revised* (DEPS-R)^(4,5)^, um questionário de autorrelato específico para detectar o risco de comportamentos alimentares desordenados em pessoas com DM1. Com uma pontuação de corte recomendada de 20 pontos ou mais, o DEPS-R facilita a avaliação clínica adicional e individualização do plano terapêutico.

No entanto, apesar da crescente atenção da comunidade científica para a temática, incluindo a construção e validação do DESP-R, ainda persiste uma lacuna significativa sobre a identificação de comportamentos alimentares desordenados em pessoas com DM1, o que evidencia escassez de evidências sistematizadas que integrem as diferentes formas de comportamentos alimentares e seus fatores associados. Essa lacuna compromete o fomento de subsídios sobre a temática, bem como a elaboração de diretrizes clínicas efetivas e a educação continuada de profissionais de saúde, a fim de reforçar a prevenção, o diagnóstico precoce e o cuidado integral de pessoas com DM1.

Nesse contexto, a condução de uma revisão de escopo se apresenta como abordagem metodológica adequada para mapear o estado atual do conhecimento, identificar lacunas, sintetizar conceitos e orientar futuras investigações. Essa abordagem é especialmente útil em áreas emergentes, como é o caso da investigação dos comportamentos alimentares desordenados em pessoas com DM1, posto que as evidências são heterogêneas, dispersas e, muitas vezes, limitadas em escopo e profundidade.

Assim, o objetivo do estudo é mapear os comportamentos alimentares desordenados em pessoas com Diabetes Mellitus tipo 1 identificados pelo instrumento DEPS-R com o intuito de identificar as lacunas sobre a interação entre os dois desfechos e apontar opções para o desenvolvimento de pesquisas futuras sobre terapêuticas baseadas em evidências, na identificação dos comportamentos alimentares desordenados associados a outros fatores.

## MÉTODO

### Desenho do Estudo

Trata-se de revisão de escopo, conduzida conforme as normas estabelecidas pelo Instituto Joanna Briggs (JBI). Desta feita, busca identificar as lacunas sobre determinada área de conhecimento. Para isso, foram consideradas as seguintes etapas para elaboração: 1) Definição e alinhamento do(s) objetivo(s) e da(s) pergunta(s); 2) Desenvolvimento e alinhamento dos critérios de inclusão com os respectivos objetivos e pergunta; 3) Descrição da abordagem planejada para busca, seleção, extração, análise e apresentação de evidências; 4) Busca das evidências; 5) Seleção das evidências; 6) Extração das evidências; 7) Análise dos resultados; 8) Apresentação dos resultados; e 9) Resumo das evidências em relação ao propósito da revisão, conclusões e implicações das descobertas^(6)^.

O protocolo desta revisão foi registrado na plataforma Open Science Framework (OSF), sob DOI: 10.17605/OSF. IO/SWNPE. Para descrição do processo de busca e seleção, por sua vez, foi utilizado o fluxograma *Preferred Reporting Items for Systematic reviews and Meta-Analyses extension for Scoping Reviews* (PRISMA-ScR)^(7)^.

### Identificação da Pergunta Norteadora

A pergunta de revisão foi estruturada a partir do mnemônico População, Conceito e Contexto (PCC), onde P diz respeito a População, ou seja, pessoas com Diabetes Mellitus tipo 1; o C; de conceito, refere-se a comportamentos alimentares desordenados; e C de contexto, à aplicação do instrumento DEPS-R para identificar os comportamentos alimentares desordenados. O presente estudo norteou-se pela seguinte pergunta norteadora: Quais são os principais comportamentos alimentares desordenados em pessoas com Diabetes Mellitus tipo 1, identificados pelo instrumento DEPS-R?

### Critérios de Elegibilidade

Foram considerados como critérios de inclusão artigos que possuem como objeto de abordagem a aplicabilidade do questionário DEPS-R para detecção de comportamentos alimentares desordenados em pessoas com DM1, publicados em qualquer idioma ou período. Foram excluídos estudos em fase de projeto, editoriais ou cartas ao editor.

### Levantamento dos Estudos

Para o vocabulário de termos controlados foram selecionados: “Diabetes mellitus type 1” e “Feeding behavior”, que integram os Descritores em Ciência da Saúde (DeCS), Medical Subject Headings (MeSH) e Thesaurus do Embase (EMTREE). Além disso, foram considerados os termos não controlados “Intrumento DEPS-R” e “DEPS-R”, junto aos operadores booleanos AND e OR, conforme disposto abaixo, no Quadro 1.

**Quadro 1:** Construção da estratégia de busca segundo mnemônico PCC – Fortaleza, CE, Brasil, 2024.

Etapas	Acrônimo		
	P	C	C
Extração	Pessoas com Diabetes Mellitus tipo 1	Comportamentos alimentares desordenados	Instrumento DEPS-R
Conversão	*Diabetes Mellitus type 1*	*Feeding behavior*	DEPS-R
Combinação	*Diabetes Mellitus type 1* *Diabetes Mellitus* *Insulin Dependent Diabetes Mellitus*	*Feeding behavior* *Eating behavior* *Feeding related behavior* *Disturbed eating behaviors*	DEPS-R
Construção	*(“Diabetes mellitus type 1” OR “Diabetes Mellitus” OR “Insulin Dependent Diabetes Mellitus”)*	*(“Feeding behavior” OR “Eating behavior” OR “Feeding related behavior” OR “Disturbed eating behaviors”)*	DEPS-R
Uso	*(“Diabetes mellitus type 1” OR “Diabetes Mellitus” OR “Insulin Dependent Diabetes Mellitus”) AND (“Feeding behavior” OR “Eating behavior” OR “Feeding related behavior” OR “Disturbed eating behaviors”) AND (DEPS-R)*		

A busca ocorreu no período de janeiro de 2024. As fontes de informações utilizadas serão PubMed (National Library of Medicine), Excerpta Medica dataBASE (EMBASE), Web of Science e Scopus. As fontes de informações foram acessadas por meio do acesso da Comunidade Acadêmica Federada (CAFe) do Portal de Periódicos da Coordenação de Aperfeiçoamento de Pessoal de Nível Superior (CAPES). A busca na literatura cinzenta contemplou o Google acadêmico e o Catálogo de Teses e Dissertações CAPES.

A seleção dos estudos ocorreu em três etapas. A primeira etapa, contemplou a identificação dos artigos, por meio da leitura do título e resumo dos artigos. A segunda etapa, por sua vez, foi a leitura na íntegra dos artigos selecionados, com o auxílio do software *Rayyan*, que controla os estudos possibilitando aos autores selecioná-los em incluídos ou excluídos nas respectivas etapas^(8)^. Nessa etapa, os autores utilizaram o duplo-cegamento para a seleção dos artigos. A fim de diminuir os conflitos de interesse, um terceiro revisor foi adicionado para decidir quais os artigos seriam eleitos para etapa de extração de dados.

Com artigos selecionados, seguiu-se para a etapa de extração dos dados e codificação dos artigos selecionados de acordo com título/codificação, autor/ano de publicação, periódico, objetivo geral do estudo, contemplando ainda o delineamento, critérios de inclusão, principais comportamentos alimentares desordenados e resultados.

### Análise e Tratamento dos Dados

Os artigos que compuseram a amostra foram organizados em uma planilha no Microsoft Excel®. Além disso, foi utilizado o *software Interface de R pour les Analyses Multidimensionnelles de Textes et de Questionnaires* (IRaMuTeQ) para análise de Similitude e Nuvem de Palavras. Este software mostra as conexões lexicais entre palavras revelando relações importantes e temas centrais dentro dos discursos.

A análise de similitude, por sua vez, representa uma rede de palavras, onde os nós (palavras centrais) representam os termos mais frequentes e relevantes nos textos analisados. As linhas, entre os nós, indicam coocorrência, ou seja, termos que aparecem frequentemente juntos. As cores dos agrupamentos sugerem temas ou subtemas relacionados. Por outro lado, a nuvem de palavras é uma representação visual da frequência com que adeterminados termos aparecem nos discursos/textos analisados. Quanto maior a palavra, maior a sua frequência ou relevância no corpus textual. Essa visualização ajuda a identificar os principais temas, preocupações e padrões linguísticos dos participantes ou documentos analisados^(9)^.

A interpretação da análise lexical foi embasada na Teoria das Representações Sociais^(10)^, a qual compreende que o conhecimento comum se forma a partir de experiências sociais compartilhadas, influenciando atitudes, práticas e percepções. Essa teoria foi particularmente útil para entender como pessoas com DM1 constroem significados em torno da imagem corporal, do uso da insulina e da alimentação.

Além disso, foram considerados referenciais específicos sobre transtornos alimentares em pessoas com DM1, como a prática de omissão da insulina com fins de emagrecimento^(11)^.

## RESULTADOS

Foram identificados 124 artigos nas bases de dados, somados aos 320 na literatura cinzenta, totalizando 424 artigos. Destes, apenas 21 artigos compuseram a amostra final, vide Figura 1.

**Figura 1:** Diagrama de fluxo PRISMA 2020 para novas revisões sistemáticas que incluíram buscas em bancos de dados e registros – Fortaleza, Ceará, Brasil, 2024.

Fonte^(7)^.

Os artigos da amostra possuem delineamento metodológico transversal, em sua maioria, sendo possível destacar medidas epidemiológicas, como a prevalência dos comportamentos alimentares desordenados nesta população, a partir de instrumento validados, a exemplo do DEPS-R. Dito isso, a organização dos principais achados, está disposta no Quadro 2, a seguir.

**Quadro 2:** Caracterização e codificação da amostra de acordo com título, autor, ano de publicação, periódico e objetivo geral – Fortaleza, Ceará, Brasil, 2024.

Título/Codificação	Autor	Periódico	Objetivo geral
*Diabetes-specific eating disorder and possible associated psychopathologies in adolescents with type 1 diabetes mellitus/A1*	Gürkan Tarçın^(12)^	Eat and Weight Disorders	Investigar a prevalência do risco de comportamentos alimentares desordenados em adolescentes com Diabetes mellitus tipo 1 e examinar as psicopatias associadas.
*The Diabetes Eating Problem Survey-Revised (DEPS-R) in a Greek Adult Population with Type 1 Diabetes Mellitus: Model Comparison Supporting a Single Factor Structure/A2*	Calliope Karastogiannidou^(13)^	Nutrients	Validar a estrutura fatorial da versão grega do DEPS-R utilizando a análise fatorial confirmatória; investigar a confiabilidade e a validade convergente da versão grega do DEPS-R e comparar um modelo DEPS-R de fator único com modelos de fatores múltiplos.
*Eating disorders between male and female adolescents with type 1 diabetes mellitus in Korea/A3*	Hye-Ryeon Park^(14)^	Belitung Nursing Journal	Identificar os fatores específicos do sexo que influenciam os distúrbios alimentares em adolescentes com Diabetes tipo 1.
*Assessment of eating disorders with the diabetes eating problems survey – revised (DEPS-R) in a representative sample of insulin-treated diabetic patients: a validation study in Italy/A4*	Frederica Pinna^(15)^	BMC Psychiatry	Avaliar em uma amostra de pacientes diabéticos tratados com insulina, as características psicométricas da versão italiana da escala DEPS-R.
*Reliability and validity of the Diabetes Eating Problem Survey in Greek adults with type 1 diabetes mellitus/A5*	Apergi^(16)^	Psychiatriki	Traduzir e adaptar o questionário DEPS-R para a língua grega, de modo a poder ser utilizado como um instrumento de rastreio curto, específico e autoadministrado para a detecção de perturbações alimentares em pessoas com Diabetes mellitus tipo 1.
*Disturbed Eating Behavior and Omission of Insulin in Adolescents Receiving Intensified Insulin Treatment: A nationwide populationbased study/A6*	Line Wisting^(17)^	Diabetes Care	Estabelecer a prevalência de distúrbios do comportamento alimentar e omissão de insulina entre adolescentes com Diabetes tipo 1 em tratamento intensivo com insulina em um estudo de base populacional de âmbito nacional.
*Associations between disordered eating behaviour, diabetes distress and emotion regulation strategies in adults with type 1 diabetes: Results from a Dutch-Italian crosssectional study/A7*	Jiska Embaye^(18)^	Diabetic medicine	Examinar as associações entre comportamentos alimentares desordenados, angústia do Diabetes e estratégias de regulação da emoção no contexto do Diabetes mellitus tipo 1.
*Psychometric properties and factor structure of the diabetes eating problem survey-revised (DEPS-R) among adults with type 1 diabetes mellitus/A8*	Yasemin Atik-Altınok^(19)^	Eat and Weight Disorders	Examinar a estrutura fatorial da versão turca do DEPS-R em adultos com DM1 e a consistência interna e a validade de construto.
*Emotional eating and disordered eating behaviors in children and adolescents with type 1 diabetes/A9*	Carlo Ripoli^(20)^	Scientific Review	Avaliar se em crianças e adolescentes com DM1 a presença de alimentação emocional está associada ao controle metabólico (hemoglobina glicada, lipídios plasmáticos, ácido úrico), com dados demográficos (idade, sexo, duração do diabetes), antropométricos (IMC-SDS), terapêuticos (método de administração de insulina, com bomba ou múltiplas injeções diárias e dose diária total) e variáveis clínicas.
*Prevalence of Eating Behaviors and Their Influence on Metabolic Control of Children with Type 1 Diabetes Mellitus/A10*	Samaneh Farnia^(21)^	Journal of Pediatrics Review	Explorar a prevalência de comportamentos alimentares e a sua influência no controle metabólico de crianças com diabetes mellitus tipo 1.
*Validation of the traditional Chinese version of the diabetes eating problem survey-revised and study of the prevalence of disordered eating patterns in Chinese patients with type 1 DM/ A11*	Lok Whi Ching^(22)^	BMC Psychiatry	Desenvolver e validar a versão tradicional chinesa do DEPS-R (C-DEPS-R), examinando a confiabilidade e a validade da escala em adultos e adolescentes com Diabetes mellitus tipo 1 que frequentavam atendimento especializado em diabetes mellitus.
*High prevalence of disordered eating behavior in Danish children and adolescents with type 1 diabetes/A12*	Francisca Nilsson^(23)^	Pediatric Diabetes	Determinar a prevalência de comportamentos alimentares desordenados em uma coorte dinamarquesa de crianças e adolescentes com Diabetes Mellitus tipo 1 com idades entre 11 e 19 anos e caracterizá-los quanto ao controle metabólico e dados clínicos relevantes.
*Translation and validation of the Diabetes Eating Problem Survey to screen eating disorders in patients with type-1 diabetes mellitus/A13*	Cintia Sancanato^(24)^	Medicine Clinic	Traduzir e validar para a população espanhola um questionário específico destinado a detectar o risco de aparecimento de um problema alimentar numa amostra de indivíduos com Diabetes Mellitus tipo 1.
*Disordered eating behaviors in adolescents with type 1 diabetes: A cross-sectional populationbased study in Italy/A14*	Valentino Cherubini^(25)^	Internacional Journal of Eating Disorders	Avaliar a associação de fatores clínicos, metabólicos e socioeconômicos com os comportamentos de transtorno alimentar entre adolescentes com diabetes tipo 1 rastreados por meio do Diabetes Eating Problem Survey-Revised (DEPS-R).
*Hypothalamic Gliosis in Adolescents with Type 1 Diabetes and Disordered Eating Behaviors/ A15*	Angel Siu Ying Nip^(26)^	Journal of the Endocrine Society	Determinar a viabilidade de avaliar a gliose hipotalâmica com ressonância magnética estrutural em adolescentes com Diabetes Mellitus tipo 1 com e sem comportamentos de transtorno alimentar.
*Disordered Eating Behaviors and Insulin Restriction in Saudi Adolescents and Young Adults with Type 1 Diabetes/A16*	Saeed Yafei^(27)^	Medicine	Estabelecer a prevalência de comportamentos alimentares desordenados e características clínicas associadas em adolescentes e adultos jovens com Diabetes Mellitus tipo 1, e o impacto dos comportamentos alimentares desordenados nos parâmetros glicêmicos.
*Disturbed Eating Behaviors in Adolescents and Emerging Adults With Type 1 Diabetes: A One-Year Prospective Study/A17*	Koen Luyckx^(28)^	Diabetes Care	Examinou os comportamentos alimentares desordenados durante um período de um ano e investigou a direcionalidade dos efeitos que ligam os comportamentos alimentares desordenados ao funcionamento específico do diabetes e aos sintomas depressivos em adolescentes e adultos emergentes.
*Brief Screening Tool for Disordered Eating in Diabetes/A18*	Markowitz^(4)^	Diabetes Care	Atualizar e validar uma ferramenta de triagem específica para diabetes para transtornos alimentares (DEPS) em jovens contemporâneos com Diabetes tipo 1.
*Prevalence of disordered eating in adults with type 1 diabetes in an Australian metropolitan hospital/A19*	Arleen Watt^(29)^	Health and social care in the Community	Determinar a prevalência de comportamentos alimentares desordenados e estabelecer os seus fatores identificáveis em adultos com Diabetes tipo 1 que frequentam um serviço hospitalar terciário.
*Disordered eating behaviours and body shape dissatisfaction among adolescents with type 1 diabetes: a cross-sectional study/A20*	Lidya Daniel^(30)^	Journal of Eating Disorders	Avaliar a magnitude dos comportamentos alimentares desordenados e a sua relação com a insatisfação com a forma do corpo entre os adolescentes com diabetes em acompanhamento.
*A Risk Profile for Disordered Eating Behaviors in Adolescents with Type 1 Diabetes: A Latent Class Analysis Study/A21*	Giada Boccolini^(31)^	Nutrients	Identificar um perfil de risco para comportamentos alimentares desordenados em jovens com diabetes tipo 1 com base na sua ingestão alimentar, perfil lipídico, índice de massa corporal e controle glico-metabólico.

DEPS-R: Diabetes Eating Problem Survey-Revised.

Os estudos destacam a presença de múltiplos comportamentos alimentares desordenados entre pessoas com DM1. Dentre estes, a omissão de insulina foi o mais prevalente^(12,13,14,15,16,17,18,19,20,21,22,23,24,25,26,27,28,29,30,31,32)^, junto ao uso inadequado de insulina^(16,17,18,19,20)^, compulsão alimentar^(12,14,20,30)^, restrição alimentar^(12,15,16,23,24)^ e comportamentos bulímicos^(22,25)^, vide Quadro 3. Adicionalmente a presença de comportamentos alimentares desordenados nessa população esteve associada a fatores relacionados a imagem corporal, como Índice de Massa Corporal (IMC) elevado, insatisfação com a imagem corporal e sintomas depressivos, de sofrimento e ansiedade. Aliado a isso, foi destacada a presença de altas taxas HbA1c e a presença de complicações decorrentes de altos níveis glicêmicos de forma precoce.

**Quadro 3:** Caracterização metodológica e principais achados de estudos sobre comportamentos alimentares desordenados em pessoas com diabetes tipo 1 – Fortaleza, Ceará, Brasil, 2024.

Codificação	Delineamento	Critérios de inclusão	Comportamentos alimentares desordenados	Resultados
A1	Estudo transversal	Adolescentes com idades entre 12 e 18 anos, com diagnóstico de diabetes tipo 1 há pelo menos um ano.	Omissão de insulina, compulsão alimentar, restrição alimentar.	O surgimento dos comportamentos alimentares desordenados está relacionado com a insatisfação da imagem corporal e índice de massa corporal Elevado.
A2	Estudo transversal	Idade superior a 18 anos e com um diagnóstico de diabetes tipo 1.	Compulsão alimentar, restrição alimentar.	A escala DEPS-R teve uma boa confiabilidade e foi positivamente associada aos níveis de hemoglobina glicada.
A3	Estudo análise secundária de dados	Pacientes com Diabetes mellitus com idade superior a 18 anos.	Omissão de insulina, compulsão alimentar.	Os comportamentos alimentares desordenados estão associados com a insatisfação com imagem corporal e depressão.
A4	Estudo transversal	Pacientes diabéticos tratados com insulina, entre 13 e 55 anos.	Omissão de insulina, restrição alimentar, comportamentos purgativos.	As mulheres apresentaram pontuações mais altas no DEPS-R, maior percentual de pelo menos um diagnóstico de Comportamento Alimentar Desordenado.
A5	Estudo transversal	Idade superior a 18 anos, com diagnóstico de diabetes tipo 1 há mais de um ano.	Compulsão alimentar, uso inadequado de insulina.	A análise fatorial revelou quatro fatores (dieta, perda de peso, uso de insulina e comportamentos compensatórios).
A6	Estudo transversal	Pessoas com diagnóstico de diabetes tipo 1 com idade de 11 a 19 anos.	Omissão de insulina, compulsão alimentar.	A presença dos comportamentos alimentares desordenados em pessoas com diabetes tipo 1 está associado com valores elevados de índice de massa corporal, hemoglobina glicada e estágio puberal.
A7	Estudo transversal	Pessoas com diagnóstico de diabetes tipo 1 com idade igual ou superior a 18 anos.	Omissão de insulina, compulsão alimentar.	A análise de percurso, com tamanhos de efeito pequenos e médios, revelou que mais angústia com a diabetes estava associada a mais transtornos alimentares.
A8	Estudo transversal	Pessoas com diagnóstico de diabetes tipo 1 com idade entre 18 e 55 anos.	Compulsão alimentar, restrição alimentar.	A análise dos resultados do DEPS-R correlacionou-se significativamente com o índice de massa corporal.
A9	Estudo transversal	Pessoas com diagnóstico de diabetes tipo 1.	Compulsão alimentar, alimentação emocional.	Indivíduos com comportamentos alimentares desordenados (DEB) apresentam pontuação alimentar emocional mais alta do que indivíduos sem DEB.
A10	Estudo transversal	Pessoas com diagnóstico de diabetes tipo 1.	Omissão de insulina, restrição alimentar.	Crianças e adolescentes apresentam comportamento de evitação das doses de insulina.
A11	Estudo transversal	Pacientes com diabetes com uso de insulina, diagnóstico nos últimos seis meses.	Omissão de insulina, compulsão alimentar.	O DEPS-R possui boa consistência. Mulheres com diabetes tipo 1 apresentam maior pontuação e nesse público os comportamentos alimentares desordenados estão associados com sintomas de depressão.
A12	Estudo transversal	Pessoas com diabetes tipo 1 com duração da doença mais de um ano.	Compulsão alimentar, restrição alimentar.	Os comportamentos alimentares desordenados estão associados com valores elevados de hemoglobina glicada e índice de massa corporal.
A13	Estudo transversal	Diagnóstico de diabetes tipo 1 de há pelo menos 12 meses, em fase de estabilidade clínica e em regime de tratamento com insulina.	Compulsão alimentar, comportamento bulímico, restrição alimentar.	A análise fatorial do DEPS-R resultou em cinco fatores: Atitudes alimentares; Comportamento bulímico; Controle de peso; Evitação; e Restrição.
A14	Estudo transversal	Diabetes tipo 1 diagnosticada pelo menos um ano antes do início do estudo.	Omissão de insulina, compulsão alimentar.	Foi identificado um perfil clínico de DEPS-R-positivo: excesso de peso, pouco tempo despendido em atividade física, baixo status socioeconômico, mau controle metabólico, saltar doses de insulina. Ademais, os jovens com excesso de peso tinham seis vezes mais probabilidades de seremDEPS-R positivos.
A15	Estudo transversal	Adolescentes com Diabetes tipo 1 com idade 13 a 19 anos.	Compulsão alimentar, omissão de insulina.	Pessoas com Diabetes Mellitus com comportamentos alimentares desordenados apresentam valores elevados de hemoglobina glicada.
A16	Estudo transversal	Adolescentes e adultos jovens sauditas com Diabetes tipo 1 (com idades entre 12 e 25 anos) em múltiplos tratamentos com insulina por pelo menos um ano.	Omissão de insulina, compulsão alimentar.	Os participantes com comportamentos alimentares desordenados apresentaram hemoglobina glicada e índice de massa corporal mais elevados em comparação com aqueles que não possuem.
A17	Estudo transversal	Jovens de língua holandesa, de 16 a 28 anos, com e sem diabetes tipo 1 comprometimento cognitivo.	Omissão de insulina, compulsão alimentar.	Pessoas com diabetes tipo 1 que possuem comportamentos alimentares desordenado possuem depressão e valores elevados de hemoglobina glicada.
A18	Estudo transversal	Adolescentes com Diabetes tipo 1 com tempo de diagnóstico de um ano.	Compulsão alimentar, omissão de insulina.	O DEPS-R apresentou correlações positivas com índice de massa corporal, conflito familiar, afeto negativo dos jovens em torno do monitoramento da glycemia.
A19	Estudo transversal	Pacientes com Diabetes tipo 1 com idade de 18 a 65 anos com diagnóstico da doença há pelo menos um ano.	Omissão de insulina, compulsão alimentar.	Mulheres com Diabetes tipo 1 apresentam valores de hemoglobina glicada e índice de massa corporal.
A20	Estudo transversal	Adolescentes com diabetes (10 a 19 anos) em acompanhamento no centro de diabetes de cinco hospitais públicos.	Compulsão alimentar, omissão de insulina.	Identificados como associação com comportamentos alimentares desordenados, fatores como insatisfação com imagem corporal, excesso de peso e complicação diabética.
A21	Estudo Multicêntrico	Adolescente com Diabetes tipo 1 e com tempo de diagnóstico de no mínimo um ano.	Compulsão alimentar, omissão de insulina.	Adolescentes que possuem risco para o desenvolvimento de comportamentos alimentares desordenados apresentam índice de massa corporal elevado.

DEPS-R: Diabetes Eating Problem Survey-Revised.

A seguir, na Figura 2A, a técnica de análise de similitude mostra as conexões lexicais entre palavras associadas a comportamentos alimentares desordenados em pessoas com DM1. Um núcleo central é destacado em grifo roxo pelos termos omissão e insulina, referindo-se à centralidade do hábito de omissão de insulina dentro dos comportamentos alimentares desordenados nessa população. Esta associação sugere indicar um comportamento de risco proposital, muitas vezes, para controle de peso, característico de transtornos alimentares como a diabulimia.

**Figura 2:** Análise de similitude (A) e nuvem de palavras (B) dos termos relacionados a comportamentos alimentares desordenados em pessoas com diabetes mellitus tipo 1.

Ademais, o grupo de palavras destacadas em grifo azul faz referência às palavras “depressivo”, “glicada” e “corporal” e “hemoglobina”, traçando uma associação entre saúde mental, como a prevalência de transtornos depressivos em pessoas com DM1, associado ao controle glicêmico (hemoglobina glicada) e imagem corporal, semelhante ao grupo verde, que aponta o perfil populacional mais vulnerável: adolescentes e jovens do sexo feminino, que estão mais propensos a desenvolverem transtornos alimentares por influência da imagem corporal e dietas. Por fim, o grupo em vermelho mostra as consequências físicas dos comportamentos alimentares desordenados: como “perda”, “peso”, “índice de massa” e “hemoglobina”.

Na Figura 2B, representação gráfica da nuvem de palavras, os termos “hemoglobina”, “corporal”, “glicada”, “insulina” e “peso” representam os eixos centrais da análise e fazem relação com tópicos como o controle glicêmico, peso corporal e imagem corporal, fortemente relacionados a transtornos alimentares, além da administração de insulina e sua possível omissão, com implicações relevantes para manutenção de comportamentos alimentares desordenados em pessoas com DM1.

## DISCUSSÃO

Os estudos indicam que indivíduos com DM1 frequentemente apresentam que a prática da diabulimia, da compulsão alimentar e da restrição revelam a complexidade do manejo nutricional nesse público, exigindo uma abordagem para além das recomendações dietéticas tradicionais. Esses comportamentos, frequentemente associados à insatisfação com a imagem corporal, sintomas depressivos e ansiedade, reforçam a necessidade de estratégias nutricionais que considerem os determinantes emocionais e psicossociais da alimentação. Além disso, a relação entre esses padrões alimentares e o controle glicêmico deficiente evidencia a relevância de intervenções nutricionais individualizadas, integradas a uma equipe multiprofissional, visando a prevenção de complicações de forma precoce nesse público.

Nesse sentido, estudos têm destacado a alta prevalência de comportamentos alimentares desordenados em pessoas com DM1, com taxas que variam de 30% a 60% dos casos, distribuídos com maior ênfase entre mulheres jovens e adolescentes^(32,33,34)^. No Brasil, estima-se que 45% dos adolescentes com DM1 apresentaram escores elevados para risco de transtornos alimentares, com associação estatisticamente significativa entre esses comportamentos e o controle glicêmico inadequado. Tais achados indicam que os comportamentos alimentares desordenados não são manifestações isoladas, mas estão profundamente integrados à experiência de viver com a DM1, influenciando tanto o bemestar físico quanto o psicológico^(35)^.

### Omissão de Insulina

A omissão de insulina é o comportamento alimentar desordenado mais comum entre pessoas com DM1, posto que a glicosúria induzida intencionalmente causa perda de peso. Em paralelo, observa-se a presença de compulsão alimentar, especialmente em momentos de estresse emocional, seguida por restrições extremas como forma de compensação. Suas causas estão associadas principalmente à consequente perda de peso. Outrossim, existem outras razões possíveis para a redução ou omissão da insulina, incluindo a ansiedade da injeção, o medo da hipoglicemia, interferência nas atividades da vida diária ou como resultado de esgotamento da diabetes devido à falta de adesão ao tratamento^(13,22,35)^.

Em mulheres jovens, a prática de omissão de insulina tem sido reportada em até 40%, de modo que esta população tem 2,4 vezes mais risco de transtorno alimentar do que mulheres da mesma idade sem DM^(36)^. Este panorama reflete a complexa interação entre os aspectos metabólicos da doença e os fatores psicossociais, questões hormonais, como preocupação com a imagem corporal, pressão estética, e insatisfação com o peso.

A puberdade e a adolescência são períodos particularmente críticos, uma vez que envolvem mudanças fisiológicas e cognitivas que intensificam a vulnerabilidade ao desenvolvimento de comportamentos disfuncionais relacionados à alimentação^(15,35)^. Além disso, fatores emocionais como ansiedade, depressão, estresse e baixa autoestima têm sido apontados como gatilhos relevantes para a manifestação desses comportamentos^(13,26)^. Assim, os comportamentos alimentares desordenados em indivíduos com DM1 representam uma condição clínica multifatorial que exige abordagem interdisciplinar e estratégias de detecção precoce.

Além disso, adolescentes estão mais propensos ao consumo de alimentos de perfil lipídico, o que colabora para o aumento do IMC, um fator de risco para o desenvolvimento dos comportamentos alimentares desordenados. Desta feita, jovens com maior tempo de diagnóstico, maior peso corporal e elevados níveis de HbA1c parecem endossar comportamentos alimentares desordenados^(22,32)^.

Nesse contexto, a neurobiologia também desempenha papel importante. O hipotálamo e o tronco cerebral são os responsáveis pela regulação do apetite e do peso. A inflamação deste órgão desempenha influência no surgimento e manutenção de comportamentos associados à alimentação, bem como comportamentos alimentares desordenados, incluindo omissão de insulina e compulsão alimentar em indivíduos com DM1^(12,18,36)^.

Estudos apontam que a distorção da imagem corporal e a busca por magreza são fatores associados à omissão de insulina como método de controle de peso, prática conhecida como diabulimia^(11,16,34)^. Essa prática pode parecer funcional no curto prazo devido à perda de peso associada à hiperglicemia induzida, mas está diretamente ligada a piores desfechos metabólicos e maior risco de complicações crônicas^(20)^. A insatisfação corporal, por sua vez, associa-se ao aumento do IMC, o que retroalimenta o ciclo de preocupação estética, omissão de insulina e desregulação alimentar^(36)^.

### Restrição e Compulsão Alimentar

Foi evidente que a insatisfação com a imagem corporal e o uso de dietas desregradas são fatores que contribuem para o surgimento de comportamentos alimentares desordenados, afetando negativamente o controle glicêmico e a saúde mental dos pacientes, bem como a prática da restrição alimentar^(13,24,26)^.

A compulsão alimentar, por vezes, integra um ciclo vicioso de dieta que envolve o consumo exagerado de alimentos com alto valor glicêmico que, quando combinados com a ausência da insulina, promove a perda de peso por meio da diurese osmótica, glicosúria, cetonúria, desidratação, permitindo ainda a excreção da energia obtida dos alimentos pela urina^(19)^.

Além da questão da imagem corporal, para alguns indivíduos, as refeições são consideradas recursos para lidar com as emoções negativas. Indivíduos com DM1 apresentam maior prevalência de depressão e ansiedade em comparação com a população geral, o que pode ser explicado tanto por fatores biológicos quanto pelo ônus psicológico do autocuidado contínuo^(13,26)^. Essa condição, entendida como alimentação emocional, está diretamente relacionada com o controle metabólico deficitário e também com o aparecimento de sintomas ansiosos e depressivos, o que culmina em episódios de compulsão alimentar seguidos por culpa e comportamentos compensatórios, como a restrição alimentar ou a omissão de insulina^(36,37)^. Estudos sugerem que altos níveis de hemoglobina glicada (HbA1c) estão associados não apenas à má adesão terapêutica, mas também à presença de sintomas depressivos e à pior percepção da imagem corporal, compondo um ciclo complexo entre saúde metabólica e saúde mental^(4,12)^.

Ademais, a carga emocional confere à compulsão alimentar a busca pelo consumo de alimentos que possam suprir sentimentos negativos vivenciados, principalmente por pessoas com idade mais jovem e IMC mais alto^(7)^. Em vista disso, a detecção precoce de comportamentos alimentares desordenados a partir do uso de questionários validados, como o DEPS-R, sugere uma avaliação mais criteriosa dos hábitos alimentares e das práticas de estilo de vida, de modo que favorece uma abordagem centrada no paciente, de modo a facilitar a construção de um vínculo terapêutico efetivo e implementação de intervenções individualizadas, ajustadas à realidade dos sujeitos^(13,15,20)^.

As pesquisas destacam que escores elevados no DEPS-R estão fortemente associados a níveis elevados de HbA1c, maior IMC e sintomas de ansiedade e depressão, evidenciando a inter-relação entre saúde mental, autopercepção corporal e adesão ao tratamento^(20,34,35,38)^. Assim, o DEPS-R representa não apenas uma ferramenta diagnóstica, mas um recurso estratégico na gestão integrada do cuidado em saúde para pessoas com DM1.

Apesar dos avanços na identificação e compreensão dos comportamentos alimentares desordenados em pessoas com DM1, ainda há importantes lacunas que precisam ser exploradas em estudos futuros. É fundamental o desenvolvimento de pesquisas longitudinais, que permitam compreender as trajetórias dos comportamentos alimentares desordenados ao longo do tempo e sua relação com o controle glicêmico, o estado emocional e os desfechos clínicos. Além disso, há carência de estudos que abordem intervenções integradas, com foco em saúde mental, educação nutricional e adesão ao tratamento, sobretudo em contextos de baixa e média renda, como o Brasil.

Ademais, investigações que utilizem abordagens qualitativas podem contribuir para uma compreensão mais profunda das experiências subjetivas e das barreiras enfrentadas por esses indivíduos no manejo da alimentação e da insulina. Nesse sentido, estudos futuros poderão fortalecer a elaboração de protocolos de rastreio precoce e intervenções terapêuticas personalizadas, promovendo um cuidado mais humanizado, efetivo e interdisciplinar às pessoas com DM1.

Destacam-se, ainda, limitações que devem ser reconhecidas. A maioria dos estudos incluídos utilizou métodos de autorrelato para avaliar os comportamentos alimentares desordenados, o que pode introduzir vieses de resposta e subnotificação dos comportamentos. Além disso, a variabilidade nos critérios de diagnóstico e nas ferramentas de avaliação entre os estudos pode ter impactado a comparabilidade dos resultados.

Outrossim, embora tenham sido adotadas abordagens sistemáticas para a inclusão dos estudos, a possibilidade de viés de seleção não pode ser completamente descartada. No entanto, essas limitações foram parcialmente mitigadas pela aplicação de critérios rigorosos de inclusão e pela análise crítica das metodologias utilizadas nos estudos selecionados. A diversidade geográfica das amostras também fortalece a generalização dos achados, proporcionando uma visão abrangente dos comportamentos alimentares desordenados em populações com DM1.

## CONCLUSÃO

O estudo evidenciou que os comportamentos alimentares desordenados são comuns em pessoas com DM1, destacando-se a omissão de insulina como o comportamento mais prevalente, seguida pela compulsão alimentar e restrição alimentar. A revisão destacou que esses comportamentos são influenciados por uma complexa interação entre fatores emocionais, cognitivos e metabólicos, como a insatisfação com a imagem corporal, além de níveis elevados HbA1c e do IMC. Esses achados sustentam a hipótese de que os comportamentos alimentares desordenados não são apenas distúrbios alimentares no sentido tradicional, mas refletem estratégias disfuncionais de enfrentamento da doença crônica, muitas vezes utilizadas como tentativa de controle do peso corporal ou de alívio emocional.

Adicionalmente, observou-se que os indivíduos mais vulneráveis a esses comportamentos são adolescentes e mulheres jovens, grupos que enfrentam maiores desafios em relação à aceitação do diagnóstico, às mudanças corporais e às exigências do tratamento intensivo com insulina. A omissão intencional da insulina, muitas vezes motivada pelo desejo de emagrecimento, pode inicialmente parecer eficaz na perda de peso, mas está associada a desfechos desfavoráveis, como o mau controle glicêmico. Esses dados ressaltam a importância de abordagens clínicas interdisciplinares, bem como estratégias de triagem precoce, como o uso do DEPS-R, para mitigar os impactos desses comportamentos na saúde física e mental de pessoas com DM1.

## DISPONIBILIDADE DE DADOS

Os dados encontram-se disponíveis no OSF: https://www.doi:10.17605/OSF.IO/SWNPE.

# Translation: Conductas alimentarias desordenadas en personas con diabetes mellitus tipo 1: revisión de alcance

**DOI:** 10.1590/1980-220X-REEUSP-2025-0177es

**Authors:** Samara Jesus Sena Marques, Thiago Santos Garces, Samuel Miranda Mattos, Lara Lídia Ventura Damasceno, Açucena Leal de Araújo, Raphaelle Sampaio Campelo Forte, Kellen Alves Freire, Virna Ribeiro Feitosa Cestari, Consuelo Helena Aires de Freitas, Thereza Maria Magalhães Moreira

**Affiliations:** 1 Universidade Estadual do Ceará, Programa de Pós Graduação em Cuidado Clínicos em Enfermagem e Saúde, Fortaleza, CE, Brasil.; 2 Universidade Estadual do Ceará, Programa de Pós-Graduação em Saúde Coletiva, Fortaleza, CE, Brasil.; 3 Universidade Regional do Cariri, Crato, CE, Brasil.; 4 Centro Universitário Maurício de Nassau, Maracanaú, CE, Brasil.

**Keywords:** Diabetes Mellitus Tipo 1, Conducta Alimentaria, Revisión de alcance

## Abstract

**Objetivo:**

Identificar los trastornos alimentarios en personas con diabetes mellitus tipo 1 a partir del instrumento *Diabetes Eating Problem Survey-Revised* (DEPS-R).

**Método:**

Se trata de una revisión de alcance, orientada por el JBI y de acuerdo con la recomendación del PRISMA ScR. Se consultaron cuatro fuentes de información y literatura gris. La búsqueda se realizó en enero de 2024. Para el análisis de similitud y la nube de palabras se utilizó el software IRaMuTeQ. La interpretación del análisis léxico se basó en la teoría de las representaciones sociales.

**Resultados:**

Los 21 artículos seleccionados describen la prevalencia de conductas alimentarias desordenadas, con especial énfasis en la omisión de insulina, la compulsión alimentaria y la restricción alimentaria. La imagen corporal, los síntomas depresivos, los altos niveles de HbA1c y la presencia de complicaciones derivadas de niveles glucémicos elevados de forma precoz también están relacionados con las conductas alimentarias desordenadas. El uso del instrumento DEPS-R demostró ser eficaz en la detección de estos comportamientos.

**Conclusión:**

Los resultados respaldan la hipótesis de que los comportamientos alimentarios desordenados no son solo trastornos alimentarios en el sentido tradicional, sino que reflejan estrategias disfuncionales para afrontar la enfermedad crónica, a menudo como un intento de controlar el peso corporal o aliviar la tensión emocional. Los estudios analíticos sobre enfoques clínicos interdisciplinarios que utilizan el instrumento DEPS-R son fundamentales para comprender la complejidad de los comportamientos alimentarios desordenados.

## INTRODUCCIÓN

El manejo intensivo de la insulinoterapia en la diabetes mellitus tipo 1 (DM1) se lleva a cabo de forma continua y exige compromiso, planificación y monitoreo constante tanto por parte del equipo multidisciplinario como de las personas con la enfermedad. Sin embargo, el control glucémico está asociado al aumento de peso, lo que favorece el desarrollo de conductas alimentarias desordenadas, definidas como hábitos alimentarios poco saludables, con el objetivo de perder peso o controlarlo. Entre ellas se incluyen la omisión de insulina, lo que se denomina diabulimia, la compulsión alimentaria, la restricción de alimentos y los métodos purgativos^(1)^.

Los principales síntomas del trastorno alimentario incluyen la búsqueda de un ideal de cuerpo excesivamente delgado, patrones alimentarios caracterizados por la restricción calórica y/o episodios de compulsión alimentaria y el uso del peso y la forma corporal para determinar la autoestima^(2)^. El panorama es aún más preocupante si se consideran los impactos de la DM1 en la autoestima y la salud mental de las personas, especialmente entre adolescentes y mujeres jóvenes, grupos más susceptibles a las preocupaciones por la imagen corporal. Las personas con DM1 tienen un mayor riesgo de desarrollar síntomas de ansiedad, depresión e insatisfacción corporal, factores que, a su vez, aumentan la vulnerabilidad a los comportamientos alimentarios desordenados^(3)^.

Para identificar estos comportamientos, se desarrolló el instrumento Diabetes Eating Problem Survey-Revised (DEPS-R)^(4,5)^, un cuestionario de autoinforme específico para detectar el riesgo de conductas alimentarias desordenadas en personas con DM1. Con una puntuación de corte recomendada de 20 puntos o más, el DEPS-R facilita la evaluación clínica adicional y la individualización del plan terapéutico.

Sin embargo, a pesar de la creciente atención de la comunidad científica hacia el tema, incluida la elaboración y validación del DESP-R, sigue existiendo una laguna significativa en la identificación de los trastornos alimentarios en personas con DM1, lo que pone de manifiesto la escasez de pruebas sistematizadas que integren las diferentes formas de conductas alimentarias y sus factores asociados. Esta laguna compromete la promoción de subsidios sobre el tema, así como la elaboración de directrices clínicas efectivas y la educación continua de los profesionales de la salud, con el fin de reforzar la prevención, el diagnóstico precoz y la atención integral de las personas con DM1.

En este contexto, la realización de una revisión de alcance se presenta como un enfoque metodológico adecuado para mapear el estado actual del conocimiento, identificar lagunas, sintetizar conceptos y orientar futuras investigaciones. Este enfoque es especialmente útil en áreas emergentes, como es el caso de la investigación de los trastornos alimentarios en personas con DM1, ya que las evidencias son heterogéneas, dispersas y, a menudo, limitadas en cuanto a su alcance y profundidad.

Por lo tanto, el objetivo del estudio es mapear los trastornos alimentarios en personas con diabetes mellitus tipo 1 identificados por el instrumento DEPS-R con el fin de identificar las lagunas sobre la interacción entre los dos resultados y señalar opciones para el desarrollo de futuras investigaciones sobre terapias basadas en la evidencia, en la identificación de trastornos alimentarios asociados a otros factores.

## MÉTODO

### Diseño del estudio

Se trata de una revisión de alcance, realizada de acuerdo con las normas establecidas por el Instituto Joanna Briggs (JBI). En este caso, busca identificar las lagunas en un área determinada del conocimiento. Para ello, se consideraron las siguientes etapas para su elaboración: 1) Definición y alineación de los objetivos y las preguntas; 2) Desarrollo y alineación de los criterios de inclusión con los respectivos objetivos y preguntas; 3) Descripción del enfoque previsto para la búsqueda, selección, extracción, análisis y presentación de las evidencias; 4) Búsqueda de las evidencias; 5) Selección de las evidencias; 6) Extracción de las evidencias; 7) Análisis de los resultados; 8) Presentación de los resultados; y 9) Resumen de las evidencias en relación con el propósito de la revisión, las conclusiones y las implicaciones de los hallazgos^(6)^.

El protocolo de esta revisión se registró en la plataforma Open Science Framework (OSF), con el DOI: 10.17605/OSF. IO/SWNPE. Para describir el proceso de búsqueda y selección, se utilizó el diagrama de flujo *Preferred Reporting Items for Systematic reviews and Meta-Analyses extension for Scoping Reviews* (PRISMA-ScR)^(7)^.

### Identificación de la Pregunta Orientadora

La pregunta de revisión se estructuró a partir del mnemotécnico Población, Concepto y Contexto (PCC), donde P se refiere a Población, es decir, personas con diabetes mellitus tipo 1; C, de concepto, se refiere a los comportamientos alimentarios desordenados; y C, de contexto, a la aplicación del instrumento DEPS-R para identificar los comportamientos alimentarios desordenados. El presente estudio se guió por la siguiente pregunta orientadora: ¿Cuáles son los principales comportamientos alimentarios desordenados en personas con diabetes mellitus tipo 1, identificados por el instrumento DEPS-R?

### Criterios de Elegibilidad

Se consideraron como criterios de inclusión los artículos que tienen como objeto de enfoque la aplicabilidad del cuestionario DEPS-R para la detección de conductas alimentarias desordenadas en personas con DM1, publicados en cualquier idioma o período. Se excluyeron los estudios en fase de proyecto, editoriales o cartas al editor.

### Recopilación de Estudios

Para el vocabulario de términos controlados se seleccionaron: “Diabetes mellitus tipo 1” y “Comportamiento alimentario”, que forman parte de los Descriptores en Ciencias de la Salud (DeCS), Medical Subject Headings (MeSH) y Thesaurus do Embase (EMTREE). Además, se consideraron los términos no controlados “Instrumento DEPS-R” y “DEPS-R”, junto con los operadores booleanos AND y OR, tal y como se muestra a continuación, en el Cuadro 1.

**Cuadro 1:** Construcción de la estrategia de búsqueda según el mnemónico PCC – Fortaleza, CE, Brasil, 2024.

Etapas	Siglas		
	P	C	C
Extracción	Personas con Diabetes Mellitus tipo 1	Comportamientos alimentarios desordenados	Instrumento DEPS-R
Conversión	*Diabetes Mellitus type 1*	*Feeding behavior*	DEPS-R
Combinación	*Diabetes Mellitus type 1* *Diabetes Mellitus* *Insulin Dependent Diabetes Mellitus*	*Feeding behavior* *Eating behavior* *Feeding related behavior* *Disturbed eating behaviors*	DEPS-R
Construcción	*(“Diabetes mellitus type 1” OR “Diabetes Mellitus” OR “Insulin Dependent Diabetes Mellitus”)*	*(“Feeding behavior” OR “Eating behavior” OR “Feeding related behavior” OR “Disturbed eating behaviors”)*	DEPS-R
Uso	*(“Diabetes mellitus type 1” OR “Diabetes Mellitus” OR “Insulin Dependent Diabetes Mellitus”) AND (“Feeding behavior” OR “Eating behavior” OR “Feeding related behavior” OR “Disturbed eating behaviors”) AND (DEPS-R)*		

La búsqueda se realizó en enero de 2024. Las fuentes de información utilizadas fueron PubMed (Biblioteca Nacional de Medicina), Excerpta Medica dataBASE (EMBASE), Web of Science y Scopus. Se accedió a las fuentes de información a través de la Comunidad Académica Federada (CAFe) del Portal de Periódicos de la Coordinación de Perfeccionamiento del Personal de Nivel Superior (CAPES). La búsqueda en la literatura gris incluyó Google Académico y el Catálogo de Tesis y Tesis Doctorales de CAPES.

La selección de los estudios se realizó en tres etapas. La primera etapa consistió en la identificación de los artículos, mediante la lectura del título y el resumen de los mismos. La segunda etapa, por su parte, consistió en la lectura íntegra de los artículos seleccionados, con la ayuda del software Rayyan, que controla los estudios y permite a los autores seleccionarlos para incluirlos o excluirlos en las respectivas etapas^(8)^. En esta etapa, los autores utilizaron el doble cegamiento para la selección de los artículos. Con el fin de reducir los conflictos de intereses, se añadió un tercer revisor para decidir qué artículos se seleccionarían para la etapa de extracción de datos.

Una vez seleccionados los artículos, se pasó a la etapa de extracción de datos y codificación de los artículos seleccionados según el título/codificación, autor/año de publicación, revista, objetivo general del estudio, contemplando también el diseño, los criterios de inclusión, los principales comportamientos alimentarios desordenados y los resultados.

### Análisis y Tratamiento de los Datos

Los artículos que componían la muestra se organizaron en una hoja de cálculo en Microsoft Excel®. Además, se utilizó el software Interface de R pour les Analyses Multidimensionnelles de Textes et de Questionnaires (IRaMuTeQ) para el análisis de similitud y nube de palabras. Este software muestra las conexiones léxicas entre las palabras, revelando relaciones importantes y temas centrales dentro de los discursos.

El análisis de similitud, por su parte, representa una red de palabras, donde los nodos (palabras centrales) representan los términos más frecuentes y relevantes en los textos analizados. Las líneas entre los nodos indican coocurrencia, es decir, términos que aparecen juntos con frecuencia. Los colores de los grupos sugieren temas o subtemas relacionados. Por otro lado, la nube de palabras es una representación visual de la frecuencia con la que aparecen determinados términos en los discursos/textos analizados. Cuanto más grande es la palabra, mayor es su frecuencia o relevancia en el corpus textual. Esta visualización ayuda a identificar los principales temas, preocupaciones y patrones lingüísticos de los participantes o documentos analizados^(9)^.

La interpretación del análisis léxico se basó en la Teoría de las Representaciones Sociales^(10)^, que entiende que el conocimiento común se forma a partir de experiencias sociales compartidas, influyendo en actitudes, prácticas y percepciones. Esta teoría fue particularmente útil para comprender cómo las personas con DM1 construyen significados en torno a la imagen corporal, el uso de la insulina y la alimentación.

Además, se consideraron referencias específicas sobre trastornos alimentarios en personas con DM1, como la práctica de omitir la insulina con fines de adelgazamiento^(11)^.

## RESULTADOS

Se identificaron 124 artículos en las bases de datos, sumados a los 320 de la literatura gris, lo que da un total de 424 artículos. De estos, solo 21 artículos formaron parte de la muestra final, véase la Figura 1.

**Figura 1:** Diagrama de flujo PRISMA 2020 para nuevas revisiones sistemáticas que incluyeron búsquedas en bases de datos y registros – Fortaleza, Ceará, Brasil, 2024.

Fuente^(7)^.

Los artículos de la muestra tienen un diseño metodológico transversal, y es posible destacar medidas epidemiológicas, como la prevalencia de conductas alimentarias desordenadas en esta población, utilizando instrumentos validados, como el DEPS-R. Dicho esto, la organización de los principales hallazgos se expone en el Cuadro 2 a continuación.

**Cuadro 2:** Caracterización y codificación de la muestra según título, autor, año de publicación, revista y objetivo general – Fortaleza, Ceará, Brasil, 2024.

Título/Codificación	Autor	Revista	Objetivo general
*Diabetes-specific eating disorder and possible associated psychopathologies in adolescents with type 1 diabetes mellitus/A1*	Gürkan Tarçın^(12)^	Eat and Weight Disorders	Investigación de la prevalencia del riesgo de conductas alimentarias desordenadas en adolescentes con diabetes mellitus tipo 1 y examen de las psicopatologías asociadas.
*The Diabetes Eating Problem Survey-Revised (DEPS-R) in a Greek Adult Population with Type 1 Diabetes Mellitus: Model Comparison Supporting a Single Factor Structure/A2*	Calliope Karastogiannidou^(13)^	Nutrients	Validar la estructura factorial de la versión griega del DEPS-R utilizando análisis factorial confirmatorio; investigar la fiabilidad y validez convergente de la versión griega del DEPS-R y comparar un modelo de factor único del DEPS-R con modelos de factores múltiples.
*Eating disorders between male and female adolescents with type 1 diabetes mellitus in Korea/A3*	Hye-Ryeon Park^(14)^	Belitung Nursing Journal	Identificar los factores específicos de género que influyen en los trastornos alimentarios en adolescentes con diabetes tipo 1.
*Assessment of eating disorders with the diabetes eating problems survey – revised (DEPS-R) in a representative sample of insulintreated diabetic patients: a validation study in Italy/A4*	Frederica Pinna^(15)^	BMC Psychiatry	Evaluar las características psicométricas de la versión italiana de la escala DEPS-R en una muestra de pacientes diabéticos tratados con insulina.
*Reliability and validity of the Diabetes Eating Problem Survey in Greek adults with type 1 diabetes mellitus/A5*	Apergi^(16)^	Psychiatriki	Traducir y adaptar el cuestionario DEPS-R al griego para que pueda ser utilizado como una herramienta de cribado breve, específica y autoadministrada para la detección de trastornos alimentarios en personas con diabetes mellitus tipo 1.
*Disturbed Eating Behavior and Omission of Insulin in Adolescents Receiving Intensified Insulin Treatment: A nationwide populationbased study/A6*	Line Wisting^(17)^	Diabetes Care	Establecer la prevalencia de trastornos de la conducta alimentaria y omisión de insulina entre adolescentes con Diabetes Tipo 1 que reciben tratamiento intensivo con insulina en un estudio de base poblacional a nivel nacional.
*Associations between disordered eating behaviour, diabetes distress and emotion regulation strategies in adults with type 1 diabetes: Results from a Dutch-Italian crosssectional study/A7*	Jiska Embaye^(18)^	Diabetic medicine	Examinar las asociaciones entre las conductas alimentarias desordenadas, la angustia diabética y las estrategias de regulación emocional en el contexto de la Diabetes mellitus tipo 1.
*Psychometric properties and factor structure of the diabetes eating problem survey-revised (DEPS-R) among adults with type 1 diabetes mellitus/A8*	Yasemin Atik-Altınok^(19)^	Eat and Weight Disorders	Examinar la estructura factorial de la versión turca del DEPS-R en adultos con DM1 y la consistencia interna y validez de constructo.
*Emotional eating and disordered eating behaviors in children and adolescents with type 1 diabetes/A9*	Carlo Ripoli^(20)^	Scientific Review	Evaluar si en niños y adolescentes con DM1 la presencia de alimentación emocional se asocia con el control metabólico (hemoglobina glicosilada, lípidos plasmáticos, ácido úrico), con datos demográficos (edad, sexo, duración de la diabetes), antropométricos (IMC-SDS), terapéuticos (método de administración de insulina, con bomba o inyecciones diarias múltiples y dosis diaria total) y variables clínicas.
*Prevalence of Eating Behaviors and Their Influence on Metabolic Control of Children with Type 1 Diabetes Mellitus/A10*	Samaneh Farnia^(21)^	Journal of Pediatrics Review	Explorar la prevalencia de las conductas alimentarias y su influencia en el control metabólico de los niños con diabetes mellitus tipo 1.
*Validation of the traditional Chinese version of the diabetes eating problem survey-revised and study of the prevalence of disordered eating patterns in Chinese patients with type 1 DM/A11*	Lok Whi Ching^(22)^	BMC Psychiatry	Desarrollar y validar la versión tradicional china de la DEPS-R (C-DEPS-R), examinando la fiabilidad y validez de la escala en adultos y adolescentes con diabetes mellitus tipo 1 que acudían a atención especializada en diabetes mellitus.
*High prevalence of disordered eating behavior in Danish children and adolescents with type 1 diabetes/A12*	Francisca Nilsson^(23)^	Pediatric Diabetes	Determinar la prevalencia de conductas alimentarias desordenadas en una cohorte danesa de niños y adolescentes con Diabetes Mellitus tipo 1 de 11–19 años y caracterizarlos en términos de control metabólico y datos clínicos relevantes.
*Translation and validation of the Diabetes Eating Problem Survey to screen eating disorders in patients with type-1 diabetes mellitus/A13*	Cintia Sancanuto^(24)^	Medicine Clinic	Traducir y validar para la población española un cuestionario específico diseñado para detectar el riesgo de padecer un problema alimentario en una muestra de individuos con Diabetes Mellitus Tipo 1.
*Disordered eating behaviors in adolescents with type 1 diabetes: A cross-sectional population-based study in Italy/A14*	Valentino Cherubini^(25)^	Internacional Journal of Eating Disorders	Evaluar la asociación de factores clínicos, metabólicos y socioeconómicos con conductas de desorden alimentario entre adolescentes con diabetes tipo 1 examinados mediante la Diabetes Eating Problem Survey-Revised (DEPS-R).
*Hypothalamic Gliosis in Adolescents with Type 1 Diabetes and Disordered Eating Behaviors/A15*	Angel Siu Ying Nip^(26)^	Journal of the Endocrine Society	Determinar la viabilidad de la evaluación de la gliosis hipotalámica con imágenes de resonancia magnética estructural en adolescentes con Diabetes Mellitus Tipo 1 con y sin conductas alimentarias desordenadas.
*Disordered Eating Behaviors and Insulin Restriction in Saudi Adolescents and Young Adults with Type 1 Diabetes/A16*	Saeed Yafei^(27)^	Medicine	Establecer la prevalencia de conductas alimentarias desordenadas y las características clínicas asociadas en adolescentes y adultos jóvenes con Diabetes Mellitus Tipo 1, y el impacto de las conductas alimentarias desordenadas en los parámetros glucémicos.
*Disturbed Eating Behaviors in Adolescents and Emerging Adults With Type 1 Diabetes: A One-Year Prospective Study/A17*	Koen Luyckx^(28)^	Diabetes Care	Se examinaron las conductas alimentarias desordenadas durante un periodo de un año y se investigó la direccionalidad de los efectos que relacionan las conductas alimentarias desordenadas con el funcionamiento específico de la diabetes y los síntomas depresivos en adolescentes y adultos emergentes.
*Brief Screening Tool for Disordered Eating in Diabetes/A18*	Markowitz^(4)^	Diabetes Care	Actualización y validación de una herramienta de cribado de trastornos alimentarios específica para la diabetes (DEPS) en jóvenes contemporáneos con diabetes tipo 1.
*Prevalence of disordered eating in adults with type 1 diabetes in an Australian metropolitan hospital/A19*	Arleen Watt^(29)^	Health and social care in the Community	Determinar la prevalencia de comportamientos alimentarios desordenados y establecer sus factores identificables en adultos con diabetes tipo 1 que acuden a un servicio hospitalario terciario.
*Disordered eating behaviours and body shape dissatisfaction among adolescents with type 1 diabetes: a cross-sectional study/A20*	Lidya Daniel^(30)^	Journal of Eating Disorders	Evaluar la magnitud de las conductas alimentarias desordenadas y su relación con la insatisfacción con la figura corporal entre adolescentes con diabetes bajo seguimiento.
*A Risk Profile for Disordered Eating Behaviors in Adolescents with Type 1 Diabetes: A Latent Class Analysis Study/A21*	Giada Boccolini^(31)^	Nutrients	Identificar un perfil de riesgo de conductas alimentarias desordenadas en jóvenes con diabetes tipo 1 basado en su ingesta dietética, perfil lipídico, índice de masa corporal y control gluco-metabólico.

DEPS-R: Encuesta de Problemas Alimentarios en la Diabetes-Revisada.

Los estudios destacan la presencia de múltiples conductas alimentarias desordenadas entre las personas con DM1. Entre ellas, la omisión de insulina fue la más prevalente^(12 13 14,15,16,17,18,19,20,21,22,23,24,25,26,27,28,29,30,31,32)^, junto con el uso inapropiado de insulina^(16,17,18,19,20)^, compulsión alimentaria^(12,14,20,30)^, restricción alimentaria^(12,15,16,23,24)^ y conductas bulímicas^(22,25)^, ver Cuadro 3. Además, la presencia de conductas alimentarias desordenadas en esta población se asoció a factores relacionados con la imagen corporal, como un Índice de Masa Corporal (IMC) elevado, insatisfacción con la imagen corporal y síntomas depresivos, sufrimiento y ansiedad.

**Cuadro 3:** Caracterización metodológica y principales hallazgos de los estudios sobre trastornos de la conducta alimentaria en personas con diabetes tipo 1 – Fortaleza, Ceará, Brasil, 2024.

Codificación	Diseño	Criterios de inclusión	Conductas alimentarias desordenadas	Resultados
A1	Estudio transversal	Adolescentes de entre 12 y 18 años, diagnosticados de diabetes tipo 1 durante al menos un año.	Omisión de insulina, compulsión alimentaria, restricción alimentaria.	La aparición de conductas alimentarias desordenadas está relacionada con la insatisfacción con la imagen corporal y un índice de masa corporal elevado.
A2	Estudio transversal	Mayores de 18 años y diagnosticados de diabetes tipo 1.	Compulsión alimentaria, restricción alimentaria.	La escala DEPS-R tuvo una buena fiabilidad y se asoció positivamente con los niveles de hemoglobina glicosilada.
A3	Estudio de análisis de datos secundarios	Pacientes con diabetes mellitus mayores de 18 años.	Omisión de insulina, compulsión alimentaria.	Las conductas alimentarias desordenadas se asocian con la insatisfacción con la imagen corporal y la depresión.
A4	Estudio transversal	Pacientes diabéticos tratados con insulina, entre 13 y 55 años.	Omisión de insulina, restricción alimentaria, conductas purgativas.	Las mujeres tenían puntuaciones DEPS-R más altas y un mayor porcentaje de al menos un diagnóstico de Conducta Alimentaria Desordenada.
A5	Estudio transversal	Mayores de 18 años, diagnosticados de diabetes tipo 1 durante más de un año.	Compulsión alimentaria, uso inadecuado de insulina.	El análisis factorial reveló cuatro factores (dieta, pérdida de peso, uso de insulina y conductas compensatorias).
A6	Estudio transversal	Personas diagnosticadas de diabetes tipo 1 de 11 a 19 años de edad.	Omisión de insulina, compulsión alimentaria.	La presencia de conductas alimentarias desordenadas en personas con diabetes tipo 1 se asocia a valores elevados de índice de masa corporal, hemoglobina glicosilada y estadio puberal.
A7	Estudio transversal	Personas diagnosticadas de diabetes tipo 1 mayores de 18 años.	Omisión de insulina, compulsión alimentaria.	El análisis de trayectorias, con tamaños del efecto pequeños y medianos, reveló que más angustia con la diabetes se asociaba con más trastornos alimentarios.
A8	Estudio transversal	Personas diagnosticadas de diabetes tipo 1 con edades comprendidas entre los 18 y los 55 años.	Compulsión alimentaria, restricción alimentaria.	El análisis de los resultados del DEPS-R correlacionó significativamente con el índice de masa corporal.
A9	Estudio transversal	Personas diagnosticadas de diabetes tipo 1.	Compulsión alimentaria, alimentación emocional.	Los individuos con conductas alimentarias desordenadas (DEB) tienen puntuaciones más altas en alimentación emocional que los individuos sin DEB.
A10	Estudio transversal	Personas diagnosticadas de diabetes tipo 1.	Omisión de insulina, restricción alimentaria.	Los niños y adolescentes muestran conductas de evitación de la dosis de insulina.
A11	Estudio transversal	Pacientes con diabetes que utilizan insulina, diagnosticados en los últimos seis meses.	Omisión de insulina, compulsión alimentaria.	El DEPS-R tiene buena consistencia. Las mujeres con diabetes tipo 1 puntuaron más alto y en este grupo las conductas alimentarias desordenadas se asocian con síntomas de depresión.
A12	Estudio transversal	Personas con diabetes tipo 1 con una duración de la enfermedad más de un año.	Compulsión alimentaria, restricción alimentaria.	Las conductas alimentarias desordenadas se asocian a valores elevados de hemoglobina glucosilada e índice de masa corporal.
A13	Estudio transversal	Diagnóstico de diabetes tipo 1 hace al menos 12 meses, en fase de estabilidad clínica y en tratamiento con insulina.	Compulsión alimentaria, comportamiento bulímico, restricción alimentaria.	El análisis factorial del DEPS-R dio como resultado cinco factores: Actitudes alimentarias; Conducta bulímica; Control del peso; Evitación; y Restricción.
A14	Estudio transversal	Diabetes tipo 1 diagnosticada al menos un año antes del inicio del estudio.	Omisión de insulina, compulsión alimentaria.	Se identificó un perfil clínico DEPS-R-positivo: sobrepeso, poco tiempo dedicado a la actividad física, estatus socioeconómico bajo, mal control metabólico, omisión de dosis de insulina. Además, los jóvenes con sobrepeso tenían seis veces más probabilidades de ser DEPS-R positivos.
A15	Estudio transversal	Adolescentes con diabetes tipo 1 de 13 a 19 años.	Compulsión alimentaria, omisión de insulina.	Las personas con diabetes mellitus con conductas alimentarias desordenadas presentan valores elevados de hemoglobina glucosilada.
A16	Estudio transversal	Adolescentes y adultos jóvenes saudíes con diabetes tipo 1 (entre 12 y 25 años) en tratamiento múltiple con insulina durante al menos un año.	Omisión de insulina, compulsión alimentaria.	Los participantes con conductas alimentarias desordenadas tenían una hemoglobina glucosilada y un índice de masa corporal más elevados en comparación con los que no las tenían.
A17	Estudio transversal	Jóvenes de habla neerlandesa, de 16 a 28 años, con y sin diabetes tipo 1 deterioro cognitivo.	Omisión de insulina, compulsión alimentaria.	Las personas con diabetes tipo 1 que presentan conductas alimentarias desordenadas tienen depresión y valores elevados de hemoglobina glucosilada.
A18	Estudio transversal	Adolescentes con diabetes tipo 1 diagnosticada en el plazo de un año.	Compulsión alimentaria, omisión de insulina.	El DEPS-R mostró correlaciones positivas con el índice de masa corporal, el conflicto familiar, el afecto negativo de los jóvenes en torno al control de la glucemia.
A19	Estudio transversal	Pacientes con diabetes tipo 1 de entre 18 y 65 años diagnosticados de la enfermedad durante al menos un año.	Omisión de insulina, compulsión alimentaria.	Mujeres con diabetes tipo 1 presentan valores de hemoglobina glicosilada e índice de masa corporal.
A20	Estudio transversal	Adolescentes con diabetes (10 a 19 años) en seguimiento en el centro de diabetes de cinco hospitales públicos.	Compulsión alimentaria, omisión de insulina.	Se identificaron factores como la insatisfacción con la imagen corporal, el exceso de peso y las complicaciones diabéticas asociados a conductas alimentarias desordenadas.
A21	Estudio multicéntrico	Adolescentes con diabetes tipo 1 y al menos un año desde el diagnóstico.	Compulsión alimentaria, omisión de insulina.	Los adolescentes con riesgo de desarrollar conductas alimentarias desordenadas tienen un índice de masa corporal elevado.

DEPS-R: Encuesta de Problemas Alimentarios en la Diabetes-Revisada.

Junto a ello, se destacó la presencia de tasas elevadas de HbA1c y la presencia de complicaciones derivadas de niveles elevados de glucemia a edades tempranas.

A continuación, en la Figura 2A, la técnica de análisis de similitud muestra las conexiones léxicas entre palabras asociadas a conductas alimentarias desordenadas en personas con DM1. Un núcleo central es resaltado en púrpura por los términos omisión e insulina, refiriéndose a la centralidad del hábito de omisión de insulina dentro de las conductas alimentarias desordenadas en esta población. Esta asociación sugiere que existe una conducta deliberada de riesgo, a menudo para controlar el peso, característica de los trastornos alimentarios como la diabulimia.

**Figura 2:** Análisis de similitud (A) y nube de palabras (B) de los términos relacionados con los trastornos alimentarios en personas con diabetes mellitus tipo 1.

Además, el grupo de palabras destacadas en azul hace referencia a las palabras “depresivo”, “glicosilado” y “corporal” y “hemoglobina”, trazando una asociación entre la salud mental, como la prevalencia de trastornos depresivos en personas con DM1, asociada al control glucémico (hemoglobina glicosilada) y la imagen corporal, similar al grupo en verde, que apunta al perfil de población más vulnerable: adolescentes y mujeres jóvenes, más propensas a desarrollar trastornos alimentarios debido a la influencia de la imagen corporal y las dietas. Por último, el grupo en rojo muestra las consecuencias físicas de las conductas alimentarias desordenadas: como “pérdida”, “peso”, “índice de masa” y “hemoglobina”.

En la Figura 2B, representación gráfica de la nube de palabras, los términos “hemoglobina”, “corporal”, “glicada”, “insulina” y “peso” representan los ejes centrales del análisis y se relacionan con temas como el control glucémico, el peso corporal y la imagen corporal, estrechamente relacionados con los trastornos alimentarios, además de la administración de insulina y su posible omisión, con implicaciones relevantes para el mantenimiento de conductas alimentarias desordenadas en personas con DM1.

## DISCUSIÓN

Los estudios indican que las personas con DM1 suelen presentar diabulimia, compulsión alimentaria y restricción, lo que revela la complejidad del manejo nutricional en este público y exige un enfoque que vaya más allá de las recomendaciones dietéticas tradicionales. Estos comportamientos, a menudo asociados con la insatisfacción con la imagen corporal, los síntomas depresivos y la ansiedad, refuerzan la necesidad de estrategias nutricionales que consideren los determinantes emocionales y psicosociales de la alimentación. Además, la relación entre estos patrones alimentarios y el control glucémico deficiente pone de manifiesto la importancia de las intervenciones nutricionales individualizadas, integradas en un equipo multidisciplinario, con el objetivo de prevenir complicaciones de forma precoz en este grupo de personas.

En este sentido, los estudios han destacado la alta prevalencia de conductas alimentarias desordenadas en personas con DM1, con tasas que varían entre el 30% y el 60% de los casos, distribuidas con mayor énfasis entre mujeres jóvenes y adolescentes^(32,34)^. En México, se estima que el 45% de los adolescentes con DM1 presentaron puntuaciones elevadas de riesgo de trastornos alimentarios, con una asociación estadísticamente significativa entre estos comportamientos y un control glucémico inadecuado. Estos hallazgos indican que los comportamientos alimentarios desordenados no son manifestaciones aisladas, sino que están profundamente integrados en la experiencia de vivir con DM1, influyendo tanto en el bienestar físico como en el psicológico^(35)^.

### Omisión de Insulina

La omisión de insulina es el comportamiento alimentario desordenado más común entre las personas con DM1, ya que la glucosuria inducida intencionalmente provoca pérdida de peso. Paralelamente, se observa la presencia de compulsión alimentaria, especialmente en momentos de estrés emocional, seguida de restricciones extremas como forma de compensación. Sus causas están asociadas principalmente a la consiguiente pérdida de peso. Además, existen otras razones posibles para la reducción u omisión de la insulina, como la ansiedad por la inyección, el miedo a la hipoglucemia, la interferencia en las actividades de la vida diaria o como resultado del agotamiento de la diabetes debido a la falta de adherencia al tratamiento^(13,22,35)^.

En las mujeres jóvenes, se ha informado que la práctica de omitir la insulina alcanza hasta un 40%, por lo que esta población tiene 2,4 veces más riesgo de trastornos alimentarios que las mujeres de la misma edad sin DM^(36)^. Este panorama refleja la compleja interacción entre los aspectos metabólicos de la enfermedad y los factores psicosociales, las cuestiones hormonales, como la preocupación por la imagen corporal, la presión estética y la insatisfacción con el peso.

La pubertad y la adolescencia son períodos particularmente críticos, ya que implican cambios fisiológicos y cognitivos que intensifican la vulnerabilidad al desarrollo de comportamientos disfuncionales relacionados con la alimentación^(15,35)^. Además, factores emocionales como la ansiedad, la depresión, el estrés y la baja autoestima se han señalado como desencadenantes relevantes para la manifestación de estos comportamientos^(13,26)^. Por lo tanto, los comportamientos alimentarios desordenados en personas con DM1 representan una condición clínica multifactorial que requiere un enfoque interdisciplinario y estrategias de detección temprana.

Además, los adolescentes son más propensos al consumo de alimentos con perfil lipídico, lo que contribuye al aumento del IMC, un factor de riesgo para el desarrollo de conductas alimentarias desordenadas. De este modo, los jóvenes con mayor tiempo de diagnóstico, mayor peso corporal y niveles elevados de HbA1c parecen respaldar las conductas alimentarias desordenadas^(22,32)^.

En este contexto, la neurobiología también desempeña un papel importante. El hipotálamo y el tronco cerebral son los responsables de la regulación del apetito y el peso. La inflamación de este órgano influye en la aparición y el mantenimiento de conductas asociadas a la alimentación, así como en conductas alimentarias desordenadas, incluyendo la omisión de insulina y la compulsión alimentaria en individuos con DM1^(12,18,36)^.

Los estudios indican que la distorsión de la imagen corporal y la búsqueda de la delgadez son factores asociados a la omisión de insulina como método de control de peso, práctica conocida como diabulimia^(11,16,34)^. Esta práctica puede parecer funcional a corto plazo debido a la pérdida de peso asociada a la hiperglucemia inducida, pero está directamente relacionada con peores resultados metabólicos y un mayor riesgo de complicaciones crónicas^(20)^. La insatisfacción corporal, a su vez, se asocia con el aumento del IMC, lo que retroalimenta el ciclo de preocupación estética, omisión de insulina y desregulación alimentaria^(36)^.

### Restricción y Compulsión Alimentaria

Ha quedado claro que la insatisfacción con la imagen corporal y el uso de dietas desequilibradas son factores que contribuyen a la aparición de comportamientos alimentarios desordenados, lo que afecta negativamente al control glucémico y a la salud mental de los pacientes, así como a la práctica de la restricción alimentaria^(13,24,26)^.

La compulsión alimentaria, a veces, forma parte de un ciclo vicioso de dieta que implica el consumo excesivo de alimentos con alto valor glucémico que, cuando se combinan con la ausencia de insulina, promueven la pérdida de peso a través de la diuresis osmótica, la glucosuria, la cetonuria y la deshidratación, lo que permite la excreción de la energía obtenida de los alimentos a través de la orina^(19)^.

Además de la cuestión de la imagen corporal, para algunas personas, las comidas se consideran recursos para lidiar con las emociones negativas. Las personas con DM1 presentan una mayor prevalencia de depresión y ansiedad en comparación con la población general, lo que puede explicarse tanto por factores biológicos como por la carga psicológica del autocuidado continuo^(13,26)^. Esta condición, conocida como alimentación emocional, está directamente relacionada con el control metabólico deficitario y también con la aparición de síntomas de ansiedad y depresión, lo que culmina en episodios de compulsión alimentaria seguidos de culpa y comportamientos compensatorios, como la restricción alimentaria o la omisión de insulina^(36 37)^. Los estudios sugieren que los niveles elevados de hemoglobina glicosilada (HbA1c) están asociados no solo a una mala adherencia terapéutica, sino también a la presencia de síntomas depresivos y a una peor percepción de la imagen corporal, lo que conforma un ciclo complejo entre la salud metabólica y la salud mental^(4,12)^.

Además, la carga emocional confiere a la compulsión alimentaria la búsqueda del consumo de alimentos que puedan suplir los sentimientos negativos experimentados, principalmente por personas más jóvenes y con un IMC más alto^(7)^. En vista de ello, la detección precoz de conductas alimentarias desordenadas mediante el uso de cuestionarios validados, como el DEPS-R, sugiere una evaluación más cuidadosa de los hábitos alimentarios y las prácticas de estilo de vida, lo que favorece un enfoque centrado en el paciente, con el fin de facilitar la construcción de un vínculo terapéutico eficaz y la implementación de intervenciones individualizadas, ajustadas a la realidad de los sujetos^(13,15,20)^.

Las investigaciones destacan que las puntuaciones elevadas en el DEPS-R están fuertemente asociadas con niveles elevados de HbA1c, mayor IMC y síntomas de ansiedad y depresión, lo que evidencia la interrelación entre la salud mental, la autopercepción corporal y la adherencia al tratamiento^(20,34 35,38)^. Así, el DEPS-R representa no solo una herramienta de diagnóstico, sino un recurso estratégico en la gestión integrada de la atención sanitaria para personas con DM1.

A pesar de los avances en la identificación y comprensión de los comportamientos alimentarios desordenados en personas con DM1, aún existen importantes lagunas que deben explorarse en futuros estudios. Es fundamental desarrollar investigaciones longitudinales que permitan comprender las trayectorias de los comportamientos alimentarios desordenados a lo largo del tiempo y su relación con el control glucémico, el estado emocional y los resultados clínicos. Además, hay una falta de estudios que aborden intervenciones integradas, centradas en la salud mental, la educación nutricional y la adherencia al tratamiento, especialmente en contextos de bajos y medianos ingresos, como Brasil.

Por otra parte, las investigaciones que utilizan enfoques cualitativos pueden contribuir a una comprensión más profunda de las experiencias subjetivas y las barreras a las que se enfrentan estas personas en el manejo de la alimentación y la insulina. En este sentido, los estudios futuros podrían reforzar la elaboración de protocolos de detección precoz e intervenciones terapéuticas personalizadas, promoviendo una atención más humanizada, eficaz e interdisciplinaria a las personas con DM1.

Cabe destacar, además, algunas limitaciones que deben reconocerse. La mayoría de los estudios incluidos utilizaron métodos de autoinforme para evaluar los comportamientos alimentarios desordenados, lo que puede introducir sesgos en las respuestas y una subnotificación de los comportamientos. Además, la variabilidad en los criterios de diagnóstico y en las herramientas de evaluación entre los estudios puede haber afectado a la comparabilidad de los resultados.

Asimismo, aunque se adoptaron enfoques sistemáticos para la inclusión de los estudios, no se puede descartar por completo la posibilidad de sesgo de selección. Sin embargo, estas limitaciones se mitigaron parcialmente mediante la aplicación de criterios de inclusión rigurosos y el análisis crítico de las metodologías utilizadas en los estudios seleccionados. La diversidad geográfica de las muestras también refuerza la generalización de los hallazgos, proporcionando una visión amplia de los comportamientos alimentarios desordenados en poblaciones con DM1.

## CONCLUSIÓN

El estudio evidenció que los trastornos alimentarios son comunes en personas con DM1, destacando la omisión de insulina como el comportamiento más prevalente, seguido por la compulsión alimentaria y la restricción alimentaria. La revisión destacó que estos comportamientos están influenciados por una compleja interacción entre factores emocionales, cognitivos y metabólicos, como la insatisfacción con la imagen corporal, además de niveles elevados de HbA1c y del IMC. Estos hallazgos respaldan la hipótesis de que los comportamientos alimentarios desordenados no son solo trastornos alimentarios en el sentido tradicional, sino que reflejan estrategias disfuncionales para afrontar la enfermedad crónica, a menudo utilizadas como un intento de controlar el peso corporal o aliviar la tensión emocional.

Además, se ha observado que las personas más vulnerables a estos comportamientos son los adolescentes y las mujeres jóvenes, grupos que se enfrentan a mayores retos en relación con la aceptación del diagnóstico, los cambios corporales y las exigencias del tratamiento intensivo con insulina. La omisión intencional de la insulina, a menudo motivada por el deseo de adelgazar, puede parecer inicialmente eficaz para perder peso, pero se asocia con resultados desfavorables, como un mal control glucémico. Estos datos resaltan la importancia de los enfoques clínicos interdisciplinarios, así como de las estrategias de detección temprana, como el uso del DEPS-R, para mitigar los impactos de estos comportamientos en la salud física y mental de las personas con DM1.

## DISPONIBILIDAD DE DATOS

Los datos están disponibles en OSF: https://www.doi:10.17605/OSF.IO/SWNPE.
